# The pervasiveness and policy consequences of medical folk wisdom in the U.S.

**DOI:** 10.1038/s41598-020-67744-6

**Published:** 2020-07-01

**Authors:** Matthew Motta, Timothy Callaghan

**Affiliations:** 1 Department of Political Science, Oklahoma State University, Stillwater, USA; 2 Department of Health Policy and Management, Texas A&M University, College Station, USA

**Keywords:** Health policy, Public health, Risk factors

## Abstract

Medical folk wisdom (MFW) refers to widely held, but factually inaccurate, beliefs about disease, immunity, pregnancy, and other medically-relevant topics. Examples include the idea that fasting when feverish (“starving a fever”) can increase the pace of recovery, or that showering after sex can prevent pregnancy. The pervasiveness of MFW, and whether or not it—like other forms of medically-relevant misinformation—shapes Americans’ health behaviors and policy preferences is an important and under-studied question. We begin this research by proposing and validating a novel measure of MFW; including a short-form scale suitable for administration in public opinion surveys. We find that nearly all Americans—irrespective of socio-economic status, political orientation, and educational background—endorse at least some aspects of MFW. Concerningly, and consistent with the idea that folk wisdom challenges scientific expertise, we additionally find that those highest in MFW tend to place less value on medical expertise and the role experts play in shaping health policy. However, this skepticism does not appear to translate to peoples’ health actions, as MFW appears to have an inconsistent effect on public participation in healthy behaviors.

## Introduction

In the age of “fake news” and the anti-vaccine movement, social scientists have taken interest in the effect of medically-relevant misinformation on Americans’ health behaviors—e.g., consulting and heeding advice from medical professionals^1^—and health policy orientations—e.g., attitudes towards universal vaccination requirements for public school children^2^. In most prior research, misinformation typically takes one of two forms: (1) *misperceptions*, or the rejection of scientific consensus on a particular issue^2–4^ (e.g., that childhood vaccines can cause autism), or (2) the endorsement of *conspiracy theories*^1,5–7^ (e.g., that pharmaceutical companies and the government conspire to create demand for unnecessary treatments).

Social scientists have made great strides in studying the effect of misinformation on health behaviors and policy attitudes. However, we believe that our understanding of medically relevant misinformation is incomplete. Specifically, we argue that while an important line of research traces the historical and anthropological origins of what we call *medical folk wisdom* (MFW)^8–11^, few have attempted to study its pervasiveness or consequences. Sometimes referred to colloquially as “old wives tales,” we define MFW as popular, yet inaccurate, information about a wide range of medically relevant topics, which are thought to have ancient origins and spread via word of mouth from parents to children^12–15^. We caution that while our conceptualization of MFW focuses on medical inaccuracy, we recognize that inaccurate beliefs may nevertheless be culturally, traditionally, or historically significant to subgroups in the population.

For example, the idea that not eating when one has a fever can speed recovery (known as “starving a fever”) can be traced back to at least the mid sixteenth century. In *A Short Dictionary for Young Beginners,* Medieval lexicographer John Withal noted that “fasting is a great remedy for a fever,” based on the idea that digesting food increases ones’ body temperature, while avoiding food can decrease it^16^. Although digestion certainly can increase body temperature, eating when sick can help replenish calories burned as the body’s immune system fights a fever. Nevertheless, more than 600 years later, the apparent pervasiveness of this belief continues to prompt debunking efforts from high profile outlets like *Scientific American,* WebMD, and the Harvard Medical School^16–18^.

Past research on MFW and related topics including folk medicine^9,10,19^ and Complementary and Alternative Medicine (CAM)^11^ has found that folk beliefs are prevalent among socio-economic and racial minorities in the US, ^12–15^ and are associated with skepticism toward advice from medical experts^12,13^. Furthermore, research has found that folk medical beliefs often rely on oral transmission, are based on coherent systems of thought and action, and vary across regions and cultural subgroups within a population^8–11,19^. Critically however, efforts to quantify the prevalence of MFW, as well as its behavioral and policy consequences *throughout* the American public are incredibly rare in previous literature^20^.

This omission is important. If MFW is in fact widespread, we might expect MFW to have a number of important implications for health policy (e.g., the rejection of expert-backed policies), and public health more generally (e.g., unwillingness to participate in healthy behaviors). Whether or not this is the case, of course, is an open empirical question.

Previous research leads us to believe that MFW could play a powerful role in shaping Americans’ health behaviors and policy attitudes. The *expert discounting hypothesis* begins with the premise that most medical folk theories, like “starving a fever,” are inconsistent with the best available scientific evidence. In Table 1, which we discuss in more detail shortly, we describe several prominent medical folk theories, and provide assessments of their accuracy. Indeed, while folk theories—like other forms of misinformation^1,4,5,7^—may have *some* elements of scientific truth, they are largely inconsistent with the best available medical evidence.

**Table 1:** Folk theory veracity assessment and endorsement summary.

Folk theory	Evidence	% Endorsed
“Exposure to cold weather can cause you to catch a cold”	Exposure to rhinoviruses, irrespective of weather conditions, causes people to catch colds^1^. People are more likely to catch colds in winter^5^, but that’s because we spend more time indoors^3^. Cold weather is associated with decreased immune responsiveness in mice^2^, and more favorable survival conditions for viruses^6^, but there is little evidence that exposure to cold weather causes colds	49% (S1), 46% (S2)
“Consuming more than the daily recommended amount of vitamin C can prevent illnesses like influenza and the common cold”	A recent meta-analysis found that vitamin C, and excess consumption of it (e.g., via supplements), does not reduce incidence of the common cold^4^	55% (S1), 49% (S2)
“Eating chicken soup can help people recover from illnesses more quickly”	Eating chicken soup may reduce respiratory inflammation, but it has no known medicinal benefits regarding its ability to fight infections^7^	66% (S1), 63% (S2)
“Not washing one’s hands can help increase immunity to disease”	Hand washing is an effective way^8^ to prevent the transfer of germs that cause disease. Poor hygiene and not washing one’s hands does not^9,10^ act as a natural form of inoculation	38% (S1), 37% (S2)
“Taking multivitamins daily can help prevent catching illnesses like the common cold”	Neither vitamin C^11^, nor multivitamins^19^ have been shown to reduce cold incidence. Some studies^12^ find beneficial effects of Zinc when taken immediately at the outset of a cold, but with the important caveat^13^ that (1) these claims are disputed, and (2) Zinc does not prevent colds	62% (S1), 72% (S2)
“Carbonated drinks, like ginger ale, can cure stomach aches”	Ginger in its natural form can have health benefits^14^. However, there is no evidence^15^ that sugar sweetened beverages like ginger ale offer much relief	63% (S1), 62% (S2)
“Women cannot become pregnant by having sex during menstruation (or “on their period”)”	While women may be less likely^16^ to become pregnant while having sex during menstruation, sex during menstruation can result in pregnancy^17^	30% (S1), 28% (S2)
“White spots on one’s fingernails are indicative of not consuming enough Vitamin C”	Although white spots (known as leukonychia) can occur for a number of different reasons, the most common is minor injury^18^ to the fingernail area. Vitamin deficiencies are not thought^20^ to be responsible for leukonychia	58% (S1), 54% (S2)
“Showering after sex is an effective way to prevent pregnancy”	Showering after sex will not^21^ help prevent pregnancy. In fact, research suggests that the act of rinsing the vagina after sex may actually push sperm further up through the cervix^22^. That said, cleaning yourself after sex can protect from some infections like UTIs^23^	15% (S1), 13% (S2)
“Cracking one’s knuckles can cause arthritis”	Consistent medical research^24,25^ suggests that cracking knuckles is not correlated with degenerative changes associated with arthritis	49% (S1), 47% (S2)
“Not eating when one has a fever (sometimes called “starving a fever”) can reduce the amount of time it takes to recover”	While the idea of fasting to stop a fever dates back to the 1500 s, it is not based in scientific consensus^26,27^. Fevers increase body temperature and metabolism and thus burn more calories. Eating can actually help to replace the calories that are lost due to the fever. That said, research in mice^28^ suggests the importance of eating may depend on the root cause of the fever, with it proving beneficial for influenza but detrimental for bacterial infections	37% (S1), 33% (S2)

For brevity and ease of interpretation, we do not include Table-specific references (in brackets) in the main text of this manuscript. A fully-referenced version of Table 1 can be found in the Supplementary information.

This incongruence creates an opportunity for those who endorse some (or many) aspects of MFW to hold negative views toward the scientific community. Negative views toward the scientific community may lead individuals to express higher levels of opposition to the role that scientific experts play in the policymaking process, and/or to be less likely to practice medically recommended health behaviors in their own lives^2^.

The precise mechanism by which expert discounting occurs could take several forms. On one hand, if people are *aware* of the scientific evidence but continue to hold these beliefs anyway, they may discount medical experts in order to alleviate cognitive dissonance^21,22^. On the other hand, if people are *unaware* of the best available scientific evidence (a “knowledge deficit”), they may subscribe to folk beliefs simply because they do not know that these views are at odds with scientific research. Although knowledge deficit models of anti-science attitudes have received important pushback in recent years^23,24^, and have difficulty explaining why people hold anti-science opinions on culturally and politically polarizing issues^25^, knowledge does play at least some role in shaping Americans’ general orientations towards science and scientific experts^26–29^.

While we are interested in assessing MFW’s policy and behavioral relevance, disentangling the precise causal mechanisms underlying these effects is outside the purview of our research. If, as some suspect^12–14^, MFW is indeed passed on generationally, answering this question ultimately requires longitudinal data that can track the formation of folk theory endorsement, as well as scientific attitudes and knowledge, over time^30^. By first finding a way to measure MFW, and characterize its pervasiveness and impact on health policy and behavior, we hope to encourage this type of data collection in the future.

## Methods

To study the pervasiveness and consequences of MFW on health policy and behavior, we fielded two opinion surveys. Study 1 (N = 509) was conducted in Spring 2019, for the purpose of offering an initial assessment of the MFW scale’s psychometric properties. Study 2 re-administered the MFW scale in a larger sample (N = 4,998), with an additional series of health policy relevant outcome variables. This research received Human Subjects approval from the Institutional Review Boards at Oklahoma State University, Yale University, the University of Pennsylvania, and Texas A&M University. All study methods were carried out in accordance with this Human Subjects approval. All participants had to be over the age of 18, and we obtained informed consent from all respondents. All data and syntax necessary to replicate this research is available for public download at the following web address: https://osf.io/v6tf8/.

Both studies recruited respondents via Lucid’s Fulcrum Academic tool, which employs quota sampling to provide samples that closely mirror national benchmarks on a variety of demographic factors (e.g., age, race, gender, education, income)^31^. Additional information about the sampling procedure, and the extent to which each sample resembles known national benchmarks, can be found in the Online Methods.

We measured MFW by asking respondents to assess whether or not eleven medical folk theories were either “definitely” or “probably” true, or “definitely” or “probably false. For example, respondents were asked whether “carbonated drinks, like ginger ale, can cure stomach aches,” and whether “cracking one’s knuckles can cause arthritis.” A list of all prompts, as well as information about their veracity and endorsement prevalence, can be found in Table 1.

We collapsed responses to each question into dichotomous indicators of whether or not respondents rated each folk theory as definitely/probably true or definitely/probably false, and combined responses into a single scale using a two parameter logistic (2PL) application of item response theory (IRT). A major benefit of this procedure—versus simply calculating the average number of theories each respondent endorsed—is that it allow items to vary in the extent to which they determine respondents’ placement on a latent MFW scale^30^. To facilitate comparison across samples, we standardize respondents’ placement on the MFW scale to range from a minimum of 0 (low endorsement) and a maximum of 1 (high endorsement).

Please note that, although we summarize the key results from the IRT procedure in some detail below, all model information is available in the Supplementary information 1. Please also consult the “Robustness check summary” section for analyses that keep each prompt in its original metric by using a graded response model application of IRT.

## Results

### The prevalence and correlates of MFW

**Figure 1:**
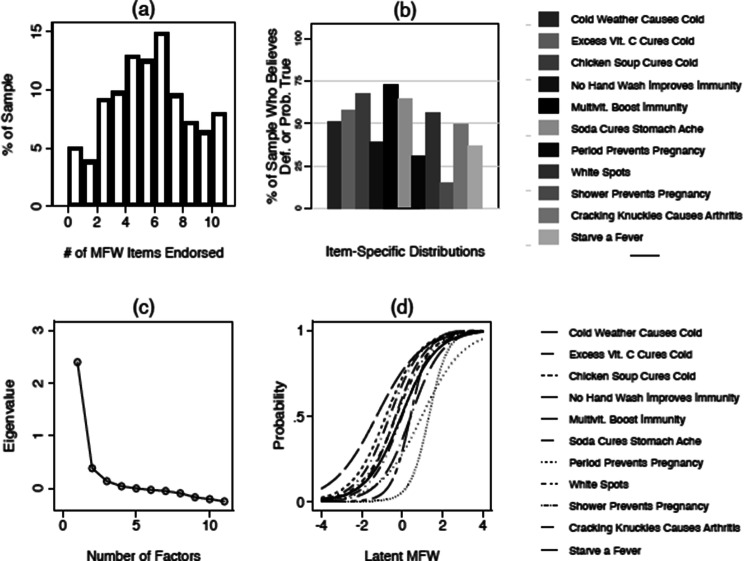
Psychometric properties of the MFW scale (Study 1). *Note* (**a**) Presents the distribution of the raw count of folk theories endorsed in the sample—i.e., the count of respondents who indicated that each theory was “definitely" or “probably" true—displayed as a histogram. (**b**) Presents percentages of respondents who endorsed each specific theory (again coded dichotomously), displayed as a bar chart. (**c**) A scree plot derived from an unrotated principal components analysis (PCA) assessing the factor structure of all 11 folk theories. The large (i.e., greater than 1) Eigenvalue associated with a one-factor solution is suggestive of unidimensionality. Finally, (**d**) plots item characteristic curves resulting from the 2PL IRT model referenced in the text. S-shaped curves indicate that people who endorse each item tend to have a high probability (y-axis) of being classified as scoring highly on the latent MFW scale (x-axis), while those who do not endorse these items tend to have a low probability of doing so.

**Figure 2:**
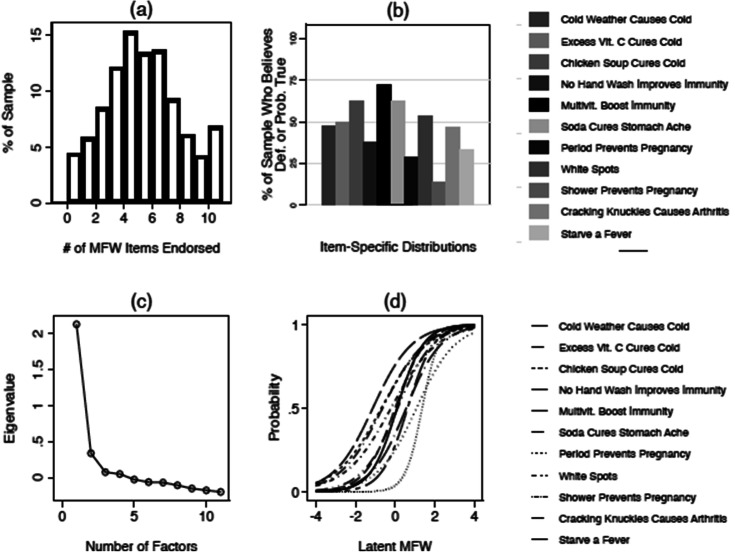
Psychometric properties of the MFW scale (Study 2). *Note* (**a**) Presents the distribution of the raw count of folk theories endorsed in the sample—i.e., the count of respondents who indicated that each theory was “definitely" or “probably" true—displayed as a histogram. (**b**) Presents percentages of respondents who endorsed each specific theory (again coded dichotomously), displayed as a bar chart. (**c**) A scree plot derived from an unrotated principal components analysis (PCA) assessing the factor structure of all 11 folk theories. The large (i.e., greater than 1) Eigenvalue associated with a one-factor solution is suggestive of unidimensionality. Finally, (**d**) plots item characteristic curves resulting from the 2PL IRT model referenced in the text. S-shaped curves indicate that people who endorse each item tend to have a high probability (y-axis) of being classified as scoring highly on the latent MFW scale (x-axis), while those who do not endorse these items tend to have a low probability of doing so.

Figures 1 and 2 provide a summary of the distribution and psychometric properties of the MFW scales administered in Study 1 and Study 2 (Figs. 1, 2, respectively). Panel a, in both figures, plots the number of specific folk theories respondents endorsed across studies. In both studies, we find that folk theory endorsement is pervasive.r The average (modal) respondent endorsed 5 out of 11 items in Study 1 (M = 5.26, SD = 2.84), and 4 out of 11 items in Study 2 (M = 4.95, SD = 2.73). Less than 6% of respondents in either study endorsed none at all, while more than 5% endorsed all 11.

Figures 1 and 2 also offer insight into *which* folk theories are the most (and least) commonly endorsed. Panel b in each figure plots mean levels of endorsement for each specific folk theory. The results document considerable variation in which theories are the most widely held. In both surveys, nearly two thirds of respondents endorsed the idea that multivitamins boost immunity to infectious disease. Many also believe that cold weather can cause the common cold (49%, S1; 47%, S2), that white spots on one’s fingernails indicate a Vitamin C deficiency (55%, S1; 53%, S2), or that abstaining from handwashing can boost immunity to infectious disease (38%, S1, 37%, S2).

Panels c and d offer a more in-depth look at the psychometrics of the MFW scale. First, some might worry that the public is more likely to endorse some folk theories (e.g., those related to immunity) relative to others (e.g., those related to sex and pregnancy); implying that MFW is not a unidimensional construct. However, principal components analyses—summarized as scree plots in Panel c—suggest that the collection of raw scores on the eleven items tend to load onto a single factor in both studies (Eigenvalue = 2.39, S1; 2.13, S2).

**Figure 3:**
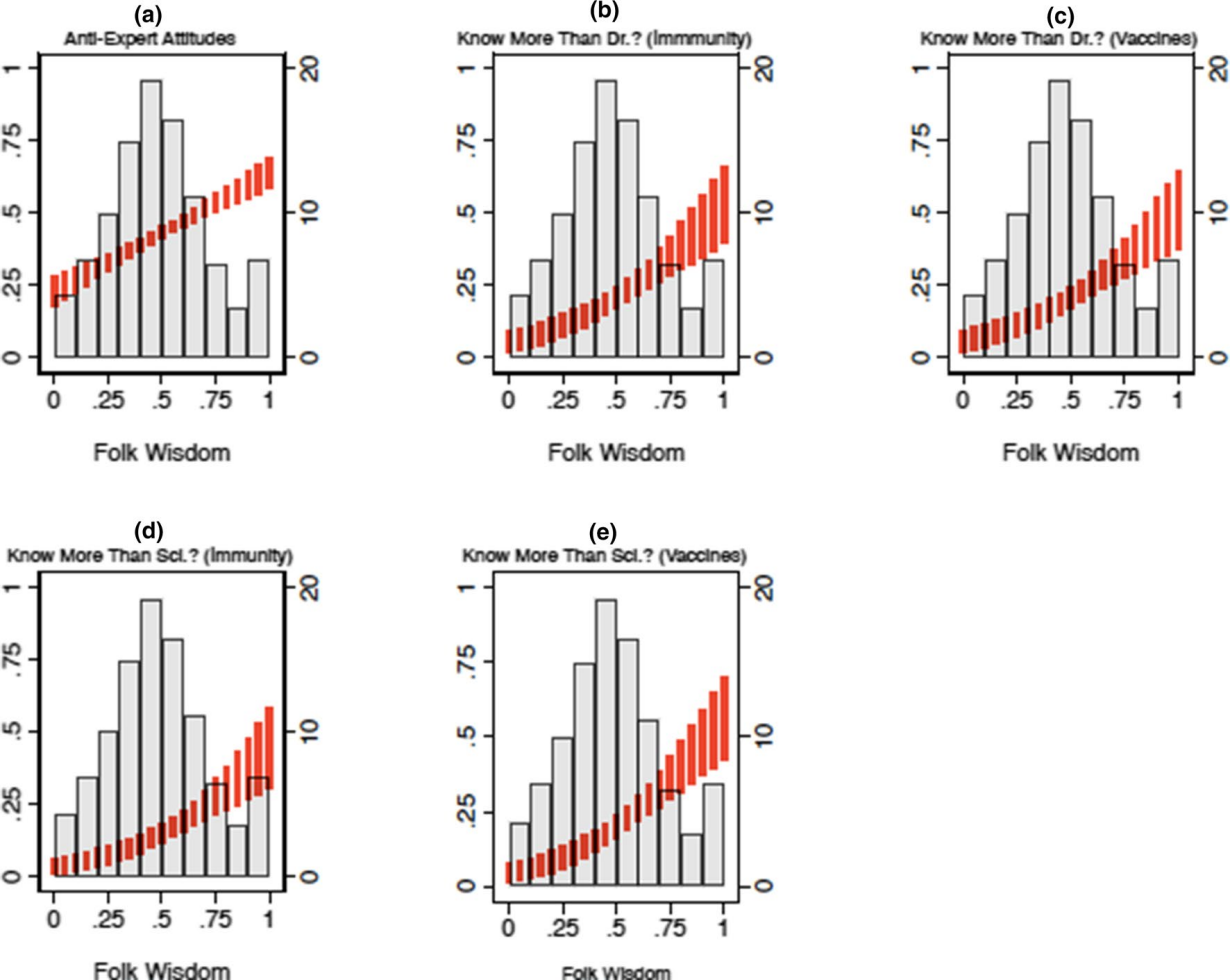
The effect of MFW on health policy attitudes (Study 1). *Note* Vertical red lines correspond to predicted values resulting from each regression model mentioned in the text, expressed as 95% confidence intervals. For reference, grayed bars correspond to the distribution of the MFW scale (derived from the IRT procedure), displayed as a histogram; with sample frequencies listed on the secondary (right-hand side) y-axis. Predicted values are linear predictions in (*a*), which displays the results of an OLS model regressing anti-expert attitude endorsement on MFW and a variety of other factors mentioned in the text. Values closer to 1 on the primary (left-hand side) y-axis indicate higher levels of negativity toward experts. Predicted values are predicted probabilities of indicating that one knows more than each respective medical expert, about each respective topic; derived from logistic regression models that regress knowledge assessments on MFW and the controls mentioned in the text. Values closer to 1 on the primary (left-hand side) y-axis indicate an increased likelihood of believing that one knows more than experts. Please consult the Supplemental information 1 for full model output.

**Figure 4:**
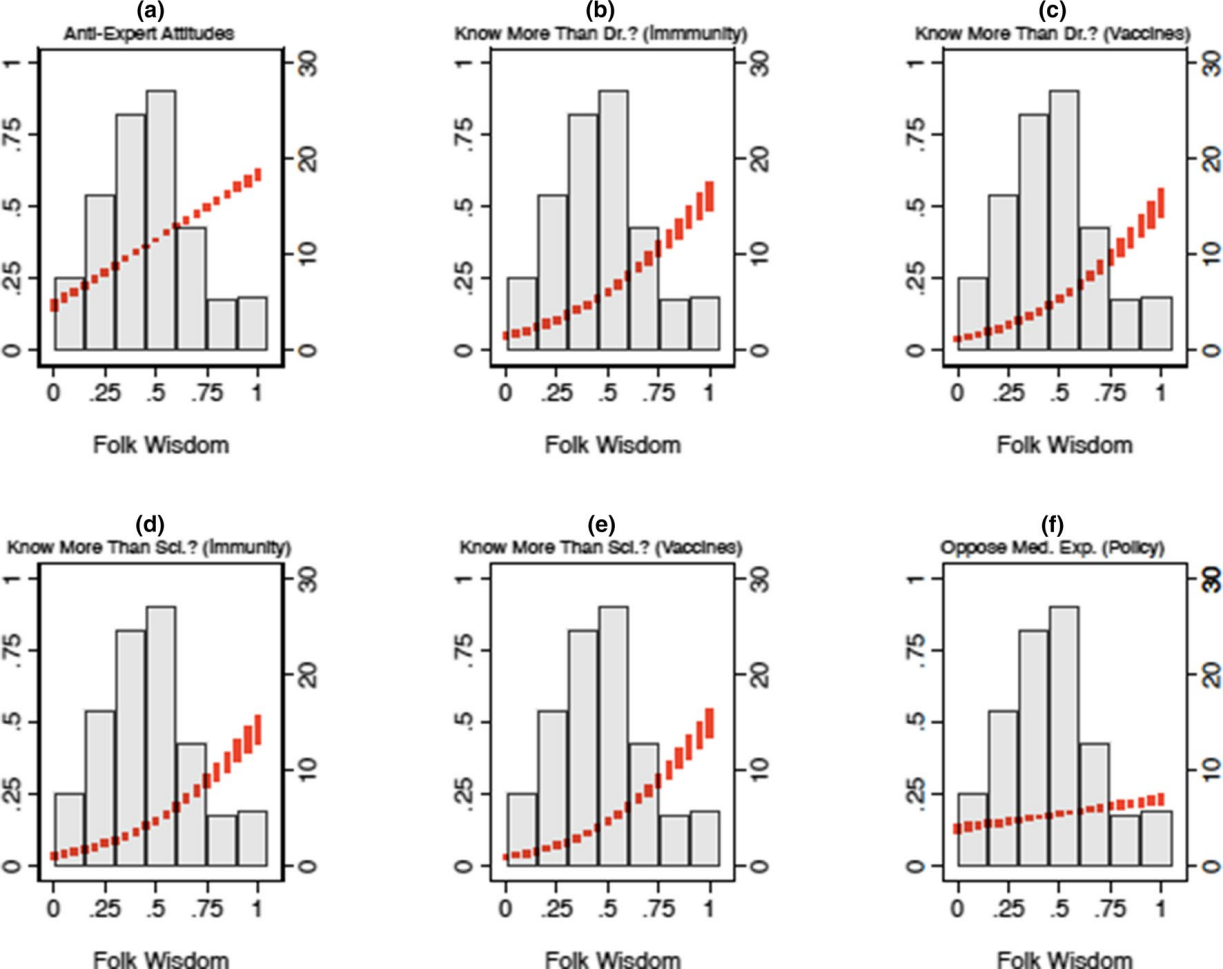
The effect of MFW on health policy attitudes (Study 2). *Note* Vertical red lines correspond to predicted values resulting from each regression model mentioned in the text, expressed as 95% confidence intervals. For reference, grayed bars correspond to the distribution of the MFW scale (derived from the IRT procedure), displayed as a histogram; with sample frequencies listed on the secondary (right-hand side) y-axis. Predicted values are linear predictions in (**a**) and (**f**), which displays the results of an OLS model regressing anti-expert attitude endorsement and opposition to the role that experts play in the policymaking process (respectively) on MFW and a variety of other factors mentioned in the text. Values closer to 1 on the primary (left-hand side) y-axis indicate higher levels of negativity toward experts. Predicted values are predicted probabilities of indicating that one knows more than each respective medical expert, about each respective topic; derived from logistic regression models that regress knowledge assessments on MFW and the controls mentioned in the text. Values closer to 1 on the primary (left-hand side) y-axis indicate an increased likelihood of believing that one knows more than experts. Please consult the Supplemental inforamtion 1 for full model output.

With strong evidence of unidimensionality in hand, we next extract a single MFW scale from these items using IRT. The scales resulting from this procedure were distributed normally (see the histograms in Figs. 3 and 4, which we discuss shortly), with a mean of about zero (M = 0.00; S1, S2) and a standard deviation of about one (SD = 0.86, S1; SD = 0.84, S2). Panel d in Figs. 1 and 2 plots item characteristic curves from the IRT models. The s-shaped curves indicate, as we should expect, that endorsement of each folk theory is associated with a sharp increase in the likelihood (y-axis) of earning a high score on the latent MFW scale (x-axis). A full list of the parameters used to build these scales can be found in the Supplemental information 1.

We further profile and validate the MFW scale by exploring its demographic and psychological correlates. In Supplementary Tables S1 and S2, we show that MFW endorsement is correlated with several factors with which we might expect it to be associated; such as low levels of objective knowledge about basic scientific facts^26–29^ and the tendency to embrace cognitive styles that prioritize feelings over facts (e.g., conspiratorial ideation)^7,32^.

### The effect of MFW on views toward health policy experts

Having demonstrated that many Americans endorse medically-relevant folk theories, we next consider the potential impact MFW might have on views toward expert-backed health policy. Specifically, Figs. 3 and 4 summarize the effect of MFW on attitudes toward medical and scientific experts, and the role that experts play in the health policymaking process.

We constructed the models used to create Figs. 3 and 4 by regressing respondents’ health expert attitudes as a function of MFW. The models account for social and political factors that could alternatively explain attitudes toward medical professionals, including: partisan identification, which, is associated with negative (for Republicans) and positive (Democrats) feelings toward expertise in general^33^; knowledge of basic scientific facts, which is moderately and positively correlated with increased levels of trust in the scientific community^23,34^ (although it may also facilitate motivated reasoning for groups skeptical of scientific expertise in certain domains^35^); and individualistic values, which may lead people to be less likely to defer to sources of expertise other than themselves. We also account for a series of demographic controls (e.g., age, race, gender, educational attainment).

Red vertical lines in each figure correspond to linear predictions or predicted probabilities—depending on the measurement of each outcome variable (which we discuss in turn)—expressed as 95% confidence intervals^35^. For reference, as noted earlier, each figure plots the distribution of the latent MFW scale in each study as a histogram (grayed bars). Full output for the models used to produce these Figures can be found in the Supplementary information 1.

Panel A in Figs. 3 and 4 presents the results of OLS models that regress agreement with two statements (e.g., “I’d rather put my trust in the wisdom of ordinary people than the opinions of experts and intellectuals”) designed to tap anti-expert sentiment^33,34^. Responses were combined into an interval-level index (alpha = 0.76; S1, S2), pursuant with prior practice^33,34^, and recoded to range from 0 to 1 (where 1 indicates holding anti-expert attitudes).

As the dashed red line in Panel A indicates, moving from the minimum to maximum observed scores on the MFW scale is associated with a steep and monotonic increase in anti-expert attitudes. Consistent with our expectations, scoring at the top of the MFW scale (relative to the bottom) is associated with more than a 40% increase in anti-expert sentiment endorsement in both Study 1 (β = 0.41, p < 0.05) and Study 2 (β = 0.45, *p* < 0.05). Note that all significance tests throughout are *two-tailed.*

Next, Panels B–E summarize the relationship between MFW and the extent to which people think of themselves as more knowledgeable than medical and scientific experts. Panels B and C asked respondents whether or not they think they know a lot more (or less), slightly more (or less), or about the same as “medical doctors” (Panel B) and “scientific researchers” (Panel C) at “preventing and treating common illnesses (like the cold or seasonal flu).” Panels D and E rely on questions structured analogously, but instead focus on respondents’ perceived knowledge about “vaccine safety and effectiveness”^2^. Responses were collapsed into binary indicators of whether or not respondents felt that they knew a lot or slightly more than medical experts—taking on a value of 1, and 0 otherwise—and modeled using logistic regression.

Again consistent with our expectations, the results show that movement from the minimum to maximum values on the MFW scale is associated with more than a 40% increase in feeling that one knows more than doctors about immunity (β = 3.25, *p* < 0.05 [S1]; β = 3.14, *p* < 0.05 [S2]) and vaccines (β = 3.07, *p* < 0.05 [S1]; β = 3.30, *p* < 0.05 [S2]). The same movement is associated with a nearly-identical increase feeling that one knows more than scientists about immunity (β = 3.42, *p* < 0.05 [S1]; β = 3.36, *p* < 0.05 [S2]) and vaccines (β = 3.65, *p* < 0.05 [S1]; β = 3.61, *p* < 0.05 [S2]), in both studies.

Finally, Panel F in Fig. 4 summarizes the effect of MFW on a series of items designed to measure respondents’ preferred role that medical experts play in the health policymaking process. Respondents were asked whether or not “medical doctors,” “scientists,” and/or the “Center for Disease Control (CDC)” should play “no role at all,” “a minor role,” or “a major role” in “making policy decisions related to public health.” For presentational simplicity, and because response patterns were similar across items, we averaged responses on these three-point scales into a single index (a = 0.70). We rescaled the index to range from 0 (indicating a preference for major roles) to 1 (indicating a preference for minor roles), and constructed the model using OLS.

Again, the results support our theoretical expectations. Moving from the minimum to maximum values on the MFW scale is associated with a 10% increase in opposition to the role that medical experts play in the policymaking process. Although these results are more modest, they are nevertheless substantively large and statistically significant (β = 0.10, *p* < 0.05).

### The effect of MFW on reported health behavior

In addition to studying the effect of MFW on respondents’ health policy orientations, we also assess whether or not those who endorse folk theories might be less likely to partake in healthy behaviors. In both studies, we asked respondents whether they “always,” “most of the time,” “just some of the time,” or “never” partake in the following; (1) “stay home from work and avoid public places when [they are] feeling sick,” (2) “wash [their] hands after using the bathroom,” (3) “wear a seatbelt when driving or riding in a car,” and (4) “visit a doctor’s office or emergency clinic when [they are] feeling sick.” Responses to each question were coded to range from 0 (never) to 3 (always), and modeled using ordered logistic regression.

In analyses available in Supplemental Tables S3 and S4, we find no evidence of a coherent relationship between MFW and healthy behavior. For example, increased MFW is associated with a decreased likelihood of regularly wearing a seatbelt, but the effect only attains conventional levels of statistical significance in Study 2 (β = − 0.58, *p* < 0.05). MFW endorsement also has no statistically discernible relationship with handwashing behavior (*p* = *n.s.*) in either study. Surprisingly, increased MFW endorsement was associated with a *positive* and statistically significant increase in the willingness to stay home (β = 0.83, *p* < 0.05 [S1]; β = 1.05, *p* < 0.05 [S2]) and visit a doctor (β = 1.54, *p* < 0.05 [S1]; β = 2.09, *p* < 0.05 [S2]) when sick. Overall, it appears that while MFW is clearly associated with negative attitudes toward the role that experts play in the policymaking process, its relationship to reported personal health behavior is both substantively and statistically unclear.

### Robustness check summary

We take several precautions to ensure that the results presented thus far are robust to plausible alternative model specifications. For example, because the individual folk theory items used to construct the MFW scale were originally administered as ordered scales, we (1) recalculated respondents’ positions on the latent MFW scale using graded response modeling (GRM see: Supplemental Tables 13, 14), and (2) re-estimated all models and figures using the updated scale (see: Supplemental Figures S1, S2 and Tables S7, S8, and S17, S18). The results are both substantively and statistically robust to this alternative specification.

Additionally, because both studies make use of survey data that are not formally representative of the American public, some might ask whether or not our assessment of the prevalence of MFW holds in more representative samples. We want to be clear that it is not our intention, in this study, to generalize specific means or frequencies from any one item, or for the entire MFW scale, to the general public. Instead, we claim that Americans’ *propensities to endorse medical folk theories* are pervasive in samples that closely (albeit imperfectly) reflect the US population.

Still, while our samples do not differ from the US adult population by much (see Table 1 in the Online Methods), our sample size in Study 2 was sufficiently large to calculate postratification survey weights. This enables us to further correct for differences between our sample and the US adult population across a range of demographic factors (e.g., age, gender, race, educational attainment, and income). Additional information about how we calculated these weights can be found in the Online Methods.

In Figure S5, we apply post-stratification weights to the results presented in Fig. 2. This procedure does not impact the number of folk theories respondents endorsed (i.e., the weighted mode remains 4 out of 11, and the mean increases slightly to M = 4.96), nor does it appear to meaningfully change item-specific frequencies. The procedure also does not influence the factor or item response structure of the MFW scale.

### A short-form MFW scale

Finally, recognizing that more work needs to be done to fully understand the origins and consequences of medical folk theory endorsement, we devised a simplified version of the MFW scale; suitable for administration in most public opinion surveys. Our short-form scale consists of just five items, which we chose on the basis of their ability to differentiate between where respondents fall on the latent MFW scale (the “discrimination parameter,” or *a*) and the relative rarity or prevalence of each item (the “difficulty parameter,” or *b*). Our goal was to select a mix of items that broadly reflect the full list on both counts.

First, we chose two items that tend to influence placement on the minima and maxima of the MFW distributions. We chose the “multivitamins boost immunity” item because it was the single most believed item (b_rank_ = 1, S1 and S2) and amongst the least discriminating (a_rank_ = 11, S1; a_rank_ = 8, S2). We also chose the “showering can prevent pregnancy” item because it was not widely believed (b_rank_ = 11, S1 and S2), but was the most discriminating (a_rank_ = 1, S1 and S2).

Next, we chose three additional items that varied in both discrimination and endorsement. We selected the “chicken soup” (b_rank_ = 2, S1 and S2; a_rank_ = 7, S1, a_rank_ = 10, S2) and “white spots” (b_rank_ = 5, S1; b_rank_ = 4, S2; a_rank_ = 8, S1; a_rank_ = 11, S2) items because both had above-average levels of endorsement, with moderate abilities to differentiate respondents’ placements on the scale. We also selected the “starve a fever” item which had below-average endorsement (b_rank_ = 8, S1; b_rank_ = 9, S2), and was highly discriminating (a_rank_ = 2, S1 and S2).

Although the items we selected for the short form scale were informed by IRT, they nevertheless require us to make subjective decisions. Consequently, we validate the scale by replicating all analyses presented above; swapping the original MFW scale for the short form version. As Figures S3, S4 and Tables S9, S10 and S19, S20 in the Supplementary information indicate, the short form scale—as might be expected^36^—introduces some amount of random measurement error into our estimates. Nevertheless, we recover substantively and statistically significant effects of MFW on health expert attitudes, and again find no consistent link between folk theory endorsement and health behavior.

## Discussion

While medically relevant misperceptions have received considerable interest from social scientists and the medical community in recent years, we argue that medical folk wisdom represents an understudied but consequential form of misinformation. Across two surveys, we find that a substantial portion of American adults endorse factually inaccurate beliefs about disease, immunity, pregnancy, and a variety of other medically-relevant topics.

Critically, we find that the endorsement of this MFW has important and negative public health consequences. Even after controlling for factors like economic status, political orientation, and educational background, Americans with higher levels of folk wisdom endorsement are less likely to value medical expertise and the important role experts play in shaping health policy. As changes in health policy are dependent on medical and scientific experts to ensure that new policies are based on the best evidence available, MFW endorsement could lead to lower levels of public support for evidence-based health policies^37^.

We also hypothesized that MFW endorsement could be associated with reported health behavior, with increased misinformation associated with fewer positive health actions. Our results however were mixed. MFW was correlated with a reduced likelihood of wearing a seatbelt, had no impact on handwashing behavior, and increased willingness to stay home or visit a doctor when sick. We suspect that we could be seeing a mixed pattern of results because even as those with high levels of MFW are motivated to reject expertise, they could nevertheless be health conscious. That is, many potential behavioral manifestations of MFW like taking multivitamins, starving a fever, or having chicken soup might reflect individuals’ drive to make healthy decisions (e.g., to improve immunity or reduce sick time). Consequently, people could be more likely to endorse MFW if they are highly concerned about their health and open to possible solutions, even if they are not supported by the medical community, in addition to exhibiting common healthy behaviors. Of course, exploring the relationship between health consciousness and MFW is beyond the scope of this project and will need to be explored in future research.

Other opportunities for additional research on MFW stand out as well. Most prominently, future longitudinal research is needed to disentangle the precise causal mechanisms connecting MFW endorsement, attitudes towards health experts, and health behaviors. While we suspect that MFW endorsement begins in early childhood as individuals are exposed to folk theories supported by their families, and that policy attitudes develop later through socialization and formative experiences in adolescence^38^, determining the causal ordering of these relationships is not possible with existing data.

In addition, while we were able to test the effects of eleven folk theories on health policy attitudes and health behavior, it is important to recognize that—although the procedure by which we measure MFW is transferrable (in both long and short form)—the specific items composing the scale itself may not be universally applicable. Folk wisdom varies across countries, cultures, and racial and ethnic groups^39–41^. It remains possible that some individuals who did not score highly on our scale could nevertheless endorse several pieces of MFW not tested through our measure. For that reason, future research should explore differences in the composition, distribution, and effects MFW across social and cultural contexts. Particular emphasis should be placed on identifying and analyzing the prevalence of folk beliefs in cultural and regional subgroups within broader populations.

Importantly, our study provides a validated short-form MFW scale to aid scholars in investigating these and other possible directions for future research. By building on our initial work here, we hope that scholars will be able to develop a broader understanding of MFW so that it can be incorporated into a better understanding of medical misinformation and its consequences.

## Supplementary information

Supplementary file1 (PDF 605 kb)

Supplementary file2 (DOCX 24 kb)
