# Supplementary material for: The pervasiveness and policy consequences of medical folk wisdom in the U.S
Source: Sci Rep. 2020 Jul 1;10:10722. doi: 10.1038/s41598-020-67744-6 (PMC7329847; doi:10.1038/s41598-020-67744-6)
Supplement: Supplementary file 1 — Supplementary file1 (PDF 605 kb) [file 41598_2020_67744_MOESM1_ESM.pdf]

The Pervasiveness and Policy Consequences of Medical Folk Wisdom in the U.S.

Matthew Motta, PhD
Assistant Professor
Department of Political Science
Oklahoma State University
matthew.motta@okstate.edu

Timothy Callaghan, PhD
Assistant Professor
Department of Health Policy & Management
Texas A&M University
callaghan@tamu.edu

Supplementary Materials: Medical Folk Wisdom

Question Wording Information **3**
- Folk Theories . . . 3
- Health Policy Outcome Variables . . . 4
  - Medical Expert Health Policy Role . . . 4
  - Anti-Expert Attitudes . . . 4
  - Comparative Expert Knowledge Assessments . . . 4
- Health Behavior Outcome Variables . . . 6
- Social, Political, & Psychological Controls . . . 7
  - Conspiratorial Ideation . . . 7
  - Individualism (Study 1) . . . 7
  - Individualism (Study 2) . . . 7
  - Health Self-Assessment . . . 8
  - Medical Doctor Access . . . 8

Main Text Analyses **9**
- Figure 1. Psychometric Properties of the MFW Scale (Study 1) . . . 9
- Figure 2. Psychometric Properties of the MFW Scale (Study 2) . . . 10
- Figure 3. The Effect of MFW on Health Policy Attitudes (Study 1) . . . 11
- Figure 4. The Effect of MFW on Health Policy Attitudes (Study 2) . . . 12

Online Methods **13**
- Methods Table 1. Comparison of Sample Characteristics to National Benchmarks (Studies 1 and 2) . . . 13

Supplementary Figures **14**
- Figure S1. Replication of Figure 3 using Graded Response Modeling . . . 14
- Figure S2. Replication of Figure 4 using Graded Response Modeling . . . 15
- Figure S3. Replication of Figure 3 using Short Form MFW Scale . . . 16
- Figure S4. Replication of Figure 4 using Short Form MFW Scale . . . 17
- Figure S5. Replication of Figure 2 with Post-stratification Weights . . . 18

Supplementary Tables **19**
- Table S1. Correlates of the MFW Scale and Individual Items (Study 1) . . . 19
- Table S2. Correlates of the MFW Scale and Individual Items (Study 2) . . . 20
- Table S3. The Effect of MFW on Health Behavior (Study 1) . . . 21
- Table S4. The Effect of MFW on Health Behavior (Study 2) . . . 22
- Table S5. Full Output for Models Used to Build Figure 3 . . . 23
- Table S6. Full Output for Models Used to Build Figure 4 . . . 24
- Table S7. Replication of Table S3 using Graded Response Modeling . . . 25
- Table S8. Replication of Table S4 using Graded Response Modeling . . . 26
- Table S9. Replication of Table S3 using Short Form MFW Scale . . . 27
- Table S10. Replication of Table S4 using Short Form MFW Scale . . . 28
- Table S11. IRT Parameters for MFW Scale: 2pl Application (Study 1) . . . 29
- Table S12. IRT Parameters for MFW Scale: 2pl Application (Study 2) . . . 30
- Table S13. IRT Parameters for MFW Scale: GRM Application (Study 1) . . . 31
- Table S14. IRT Parameters for MFW Scale: GRM Application (Study 2) . . . 33
- Table S15. IRT Parameters for Short Form MFW Scale (Study 1) . . . 35
- Table S16. IRT Parameters for Short Form MFW Scale (Study 2) . . . 36
- Table S17. Models Used to Produce Figure S1 . . . 37
- Table S18. Models Used to Produce Figure S2 . . . 38
- Table S19. Models Used to Produce Figure S3 . . . 39
- Table S20. Models Used to Produce Figure S4 . . . 40

Summary Statistics **41**
- Table S21. Study 2 Summary Statistics (for Variables NOT Presented in Table M1) . . . 41
- Table S22. Study 2 Summary Statistics (for Variables NOT Presented in Table M1) . . . 42

Question Wording Information

Note: items were administered in both Studies 1 and 2 (except where otherwise noted), and worded identically in both cases. Please consult the main text and/or Online Data for additional information about how the coding and scaling of these items.

Folk Theories

Please note that all items are presented **in random order**. Also note that items 4 and 5 are NOT folk theories (i.e., they are factually correct), as to avoid presenting respondents with only inaccurate statements. Recall that information about the coding and scaling of these items can be found in the main text.

```
PREAMBLE. Please read the following series of statements. To the best of your knowledge, please
tell us whether or not you think each one is definitely true, probably true, probably not true,
or definitely not true.

1. Exposure to cold weather can cause you to catch a cold.

2. Consuming more than the daily recommended amount of vitamin C can prevent illnesses
like influenza and the common cold.

3. Eating chicken soup can help people recover from illnesses more quickly.

4. Illnesses like the common cold are primarily caused by microscopic organisms ("germs")

5. Washing one's hands can help stop the spread of disease [Not included: see note above]

6. Not washing ones hands can help increase immunity to disease [Not included: see note above]

7. Taking multivitamins daily can help prevent catching illnesses like the common cold.

8. Carbonated drinks, like ginger ale, can cure stomach aches

9. Women cannot become pregnant by having sex during menstruation (or on their period).

10. White spots on ones fingernails are indicative of not consuming enough Vitamin C.

11. Showering after sex is an effective way to prevent pregnancy.

12. Cracking ones knuckles can cause arthritis.

13. Not eating when one has a fever (sometimes called "starving a fever")
can reduce the amount of time it takes to recover
```

Health Policy Outcome Variables

Medical Expert Health Policy Role

Note that these items were only administered in Study 2. Starred items were included in analyses presented in the main text. All items were presented in random order.

Please tell us whether you think each of the following groups should play a major role, a minor role, or no role at all in making policy decisions related to public health.

1. * Medical Doctors

2. * The Center for Disease Control (CDC)

3. * Scientists

4. Congress

5. Parents

6. The President of the United States

<1> A major role
<2> A minor role
<3> No role at all

Anti-Expert Attitudes

Note that item #3 is not included in the scale presented in the main text, as doing so decreases inter-item reliability (from 0.76 to 0.59 in Study 1, and from 0.76 to 0.58 in Study 2).

Please read the following statements, and tell us the extent to which you agree or disagree with each one.

1. I'd rather put my trust in the wisdom of ordinary people than the opinions of experts and intellectuals.

2. When it comes to really important questions, scientific facts don't help very much

3. Ordinary people can really use the help of experts to understand complicated things like science and health

<1> Strongly agree
<2> Agree
<3> Somewhat agree
<4> Neither agree nor disagree
<5> Somewhat disagree
<6> Disagree
<7> Strongly disagree

Comparative Expert Knowledge Assessments

Note, all items – as well as collections of items (i.e., knowledge about vaccine safety vs. knowledge about infectious disease) – were presented in random order. Starred items – which pertain to more-general references of medical/scientific professionals (as opposed to specific institutions) – were included in our analyses.

VACCINE SAFETY KNOWLEDGE

Compared to each of the following groups, would you say that you know a lot more, slightly more,

about the same, slightly less, or a lot less about vaccine safety and effectiveness?

1. * Medical doctors

2. The Center for Disease Control (CDC)

3. Public health officials in your state

4. * Scientific researchers

<1> I know a lot more
<2> I know slightly more
<3> I know about the same
<4> I know slightly less
<5> I know a lot less

INFECTIOUS DISEASE KNOWLEDGE

Compared to each of the following groups, would you say that you know a lot more, slightly more, about the same, slightly less, or a lot less about preventing and treating common illnesses (like the cold or seasonal flu)?

1. * Medical doctors

2. The Center for Disease Control (CDC)

3. Public health officials in your state

4. * Scientific researchers

Health Behavior Outcome Variables

Please note that all items were presented in random order.

```
Please tell us how often you do each of the following.

1. Stay home from work and avoid public places when you are feeling sick

2. Wash your hands after using the bathroom

3. Wear a seatbelt when driving or riding in a car

4. Visit a doctor's office or emergency care clinic when you are feeling sick

<1> Always
<2> Most of the time
<3> Just some of the time
<4> Never
```

Social, Political, & Psychological Controls

Conspiratorial Ideation

Please note that, due to survey administration constraints, conspiratorial ideation was administered in Study 1 only.

```
Please tell us whether you agree or disagree with the following statements.

1. Events like wars, recessions, and the outcomes of elections are controlled by small
groups of people who are working in secret against the rest of us.

2. Much of our lives are being controlled by plots hatched in secret places

3. Even though we live in a democracy, a few people will always run things anyway

4. The people who really 'run' the country are not known to the voters

<1> Strongly agree
<2> Agree
<3> Somewhat agree
<4> Neither agree nor disagree
<5> Somewhat disagree
<6> Disagree
<7> Strongly disagree
```

Individualism (Study 1)

Please note that we measured individualism in Study 1 using the individualism dimension of the Cultural Cognition inventory (see: Kahan et al., 2012; https://papers.ssrn.com/sol3/papers.cfm?abstract_id=2193133). Items denoted with [R] were reverse coded.

```
People in our society often disagree about how far to let individuals go in making decisions for
themselves. How strongly you agree or disagree with each of these statements?

1. The government interferes far too much in our everyday lives.

2. [R] Sometimes government needs to make laws that keep people from hurting themselves.

3. It's not the government's business to try to protect people from themselves.

4. The government should stop telling people how to live their lives.

5. [R] The government should do more to advance society's goals, even if that means limiting
the freedom and choices of individuals.

6. [R] Government should put limits on the choices individuals can make so they don't
 get in the way of what's good for society.
```

Individualism (Study 2)

Please note that starred items are used to measure Self Enhancement values (our measure of individualism in Study 2) from Shalom Schwartz's Basic Human Value Inventory (see: https://scholarworks.gvsu.edu/cgi/viewcontent.cgi?article=1116&context=orpc). Items were presented in random order.

```
Please tell us the extent to which you agree or disagree with each of the following statements.
```

1. It is important to me to be rich. I want to have a lot of money and expensive things.

2. It is important to me to be in charge and tell others what to do. I want people to do what I say.

3. It is very important to show others my abilities. I want people to admire what I do.

4. Being very successful is important to me. I like to impress other people.

<1> Strongly disagree
<2> Disagree
<3> Somewhat disagree
<4> Neither agree nor disagree
<5> Somewhat agree
<6> Agree
<7> Strongly agree

Health Self-Assessment

Would you say that in general your health is...
<1> Excellent
<2> Very good
<3> Good
<4> Fair
<5> Poor

Medical Doctor Access

Was there a time in the past 12 months when you needed to see a doctor but could not because of transportation issues?

<1> Yes
<2> No

Main Text Analyses

Figure 1. Psychometric Properties of the MFW Scale (Study 1)

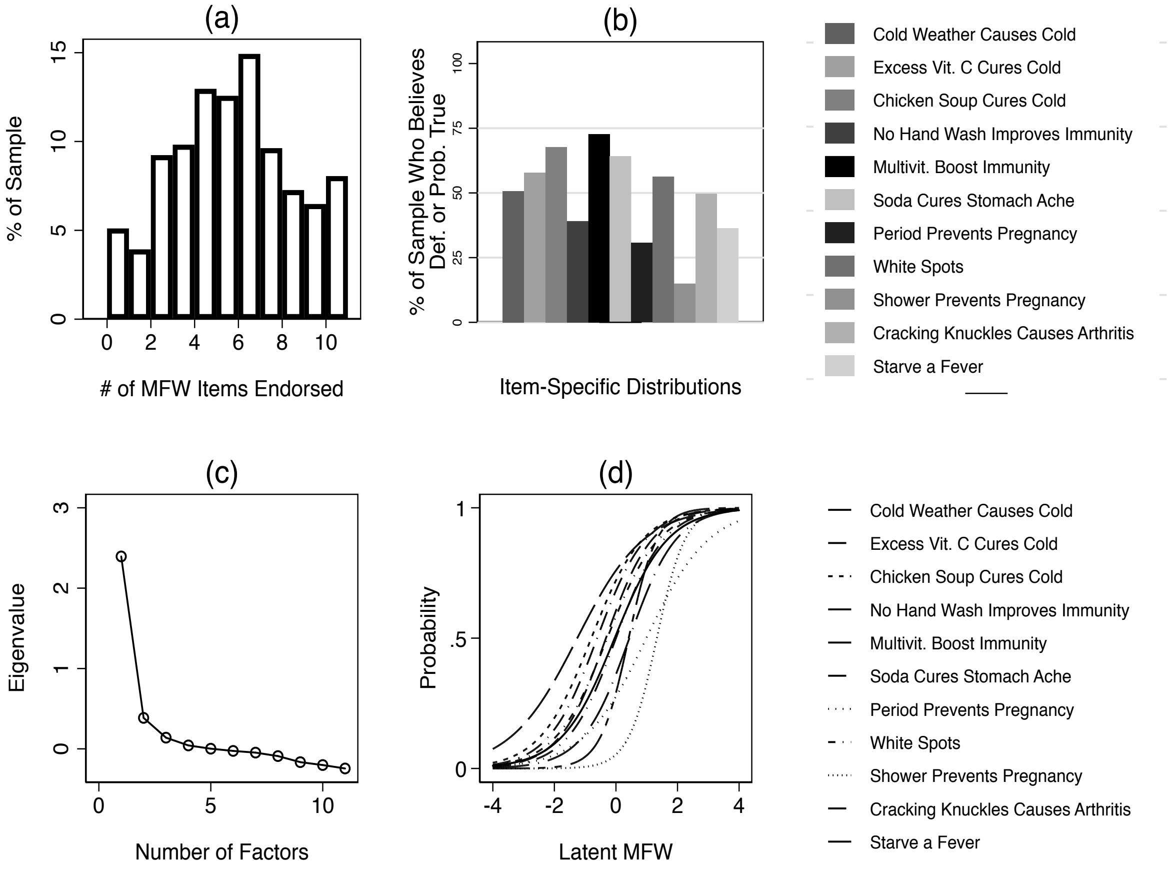

Note. Panel (a) presents the distribution of the raw count of folk theories endorsed in the sample – i.e., the count of respondents who indicated that each theory was "definitely" or "probably" true – displayed as a histogram. Panel (b) presents percentages of respondents who endorsed each specific theory (again coded dichotomously), displayed as a bar chart. Panel (c) is a scree plot derived from an unrotated principal components analysis (PCA) assessing the factor structure of all 11 folk theories. The large (i.e., greater than 1) Eigenvalue associated with a one-factor solution is suggestive of unidimensionality. Finally, panel (d) plots item characteristic curves resulting from the 2PL IRT model referenced in the text. S-shaped curves indicate that people who endorse each item tend to have a high probability (y-axis) of being classified as scoring highly on the latent MFW scale (x-axis), while those who do not endorse these items tend to have a low probability of doing so.

Figure 2. Psychometric Properties of the MFW Scale (Study 2)

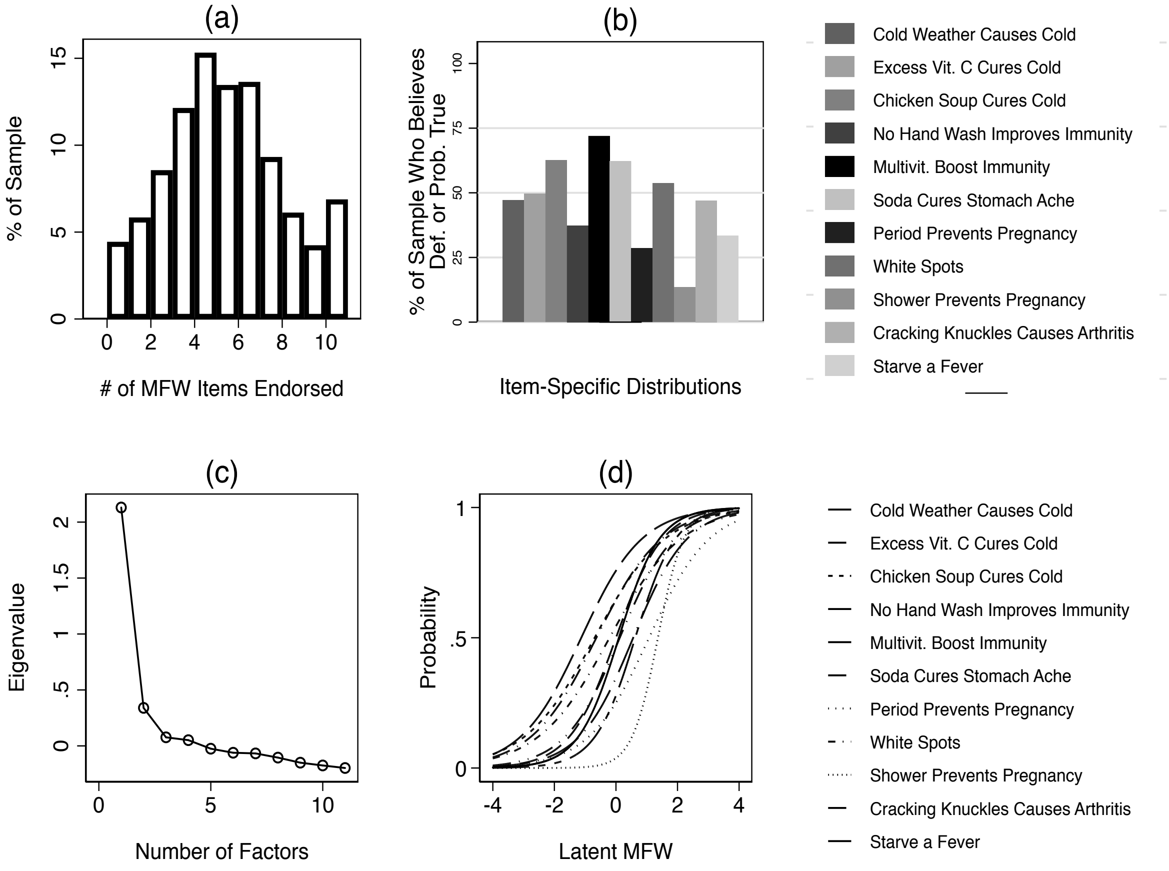

Note. Panel (a) presents the distribution of the raw count of folk theories endorsed in the sample – i.e., the count of respondents who indicated that each theory was “definitely” or “probably” true – displayed as a histogram. Panel (b) presents percentages of respondents who endorsed each specific theory (again coded dichotomously), displayed as a bar chart. Panel (c) is a scree plot derived from an unrotated principal components analysis (PCA) assessing the factor structure of all 11 folk theories. The large (i.e., greater than 1) Eigenvalue associated with a one-factor solution is suggestive of unidimensionality. Finally, panel (d) plots item characteristic curves resulting from the 2PL IRT model referenced in the text. S-shaped curves indicate that people who endorse each item tend to have a high probability (y-axis) of being classified as scoring highly on the latent MFW scale (x-axis), while those who do not endorse these items tend to have a low probability of doing so.

Figure 3. The Effect of MFW on Health Policy Attitudes (Study 1)

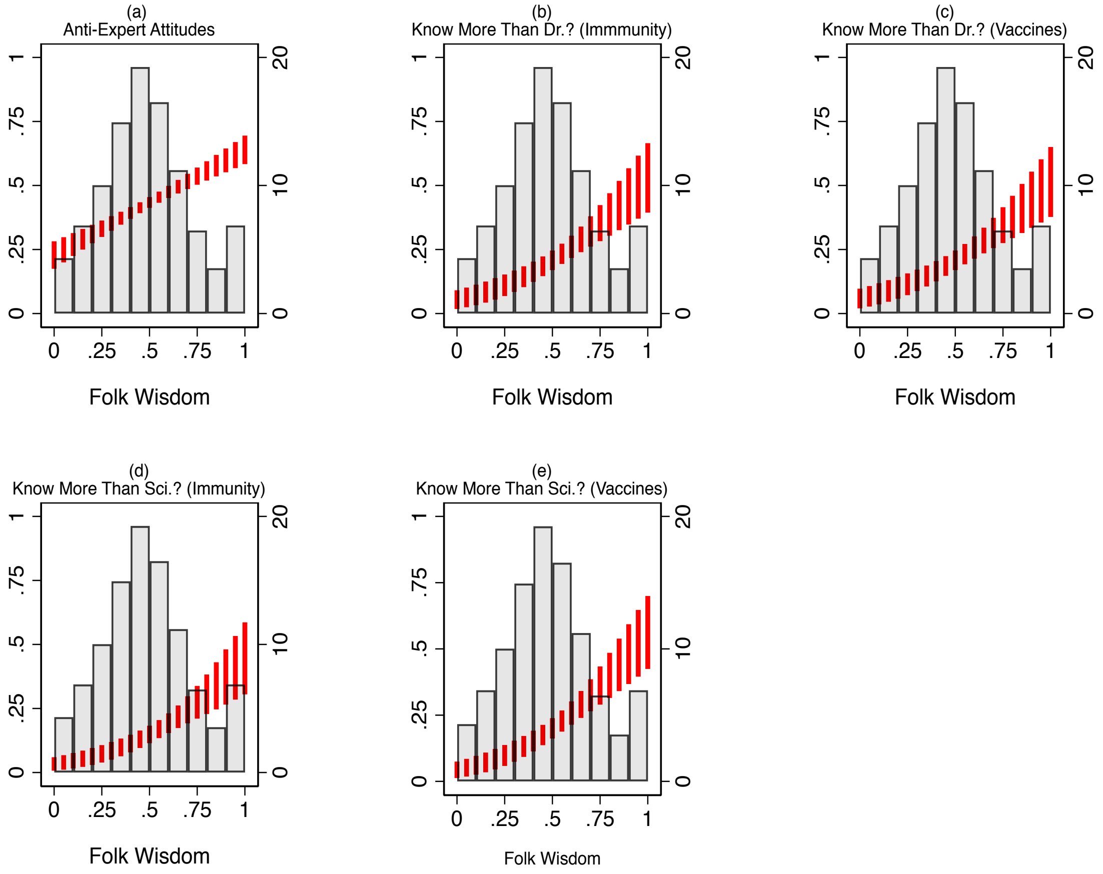

Note. Vertical red lines correspond to predicted values resulting from each regression model mentioned in the text, expressed as 95% confidence intervals. For reference, grayed bars correspond to the distribution of the MFW scale (derived from the IRT procedure), displayed as a histogram; with sample frequencies listed on the secondary (right-hand side) y-axis. Predicted values are linear predictions in (a), which displays the results of an OLS model regressing anti-expert attitude endorsement on MFW and a variety of other factors mentioned in the text. Values closer to 1 on the primary (left-hand side) y-axis indicate higher levels of negativity toward experts. Predicted values are predicted probabilities of indicating that one knows more than each respective medical expert, about each respective topic; derived from logistic regression models that regress knowledge assessments on MFW and the controls mentioned in the text. Values closer to 1 on the primary (left-hand side) y-axis indicate an increased likelihood of believing that one knows more than experts. Please consult the Supplemental Materials for full model output.

Figure 4. The Effect of MFW on Health Policy Attitudes (Study 2)

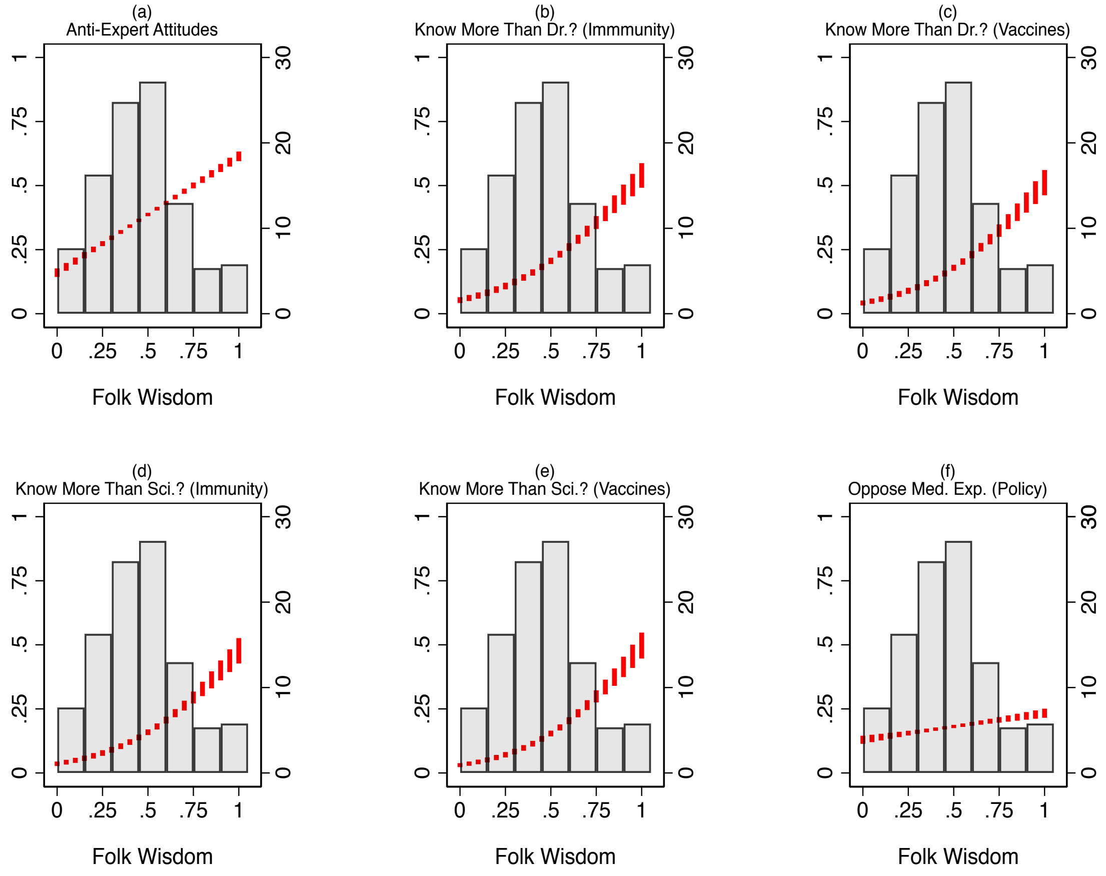

Note. Vertical red lines correspond to predicted values resulting from each regression model mentioned in the text, expressed as 95% confidence intervals. For reference, grayed bars correspond to the distribution of the MFW scale (derived from the IRT procedure), displayed as a histogram; with sample frequencies listed on the secondary (right-hand side) y-axis. Predicted values are linear predictions in (a) and (f), which displays the results of an OLS model regressing anti-expert attitude endorsement and opposition to the role that experts play in the policymaking process (respectively) on MFW and a variety of other factors mentioned in the text. Values closer to 1 on the primary (left-hand side) y-axis indicate higher levels of negativity toward experts. Predicted values are predicted probabilities of indicating that one knows more than each respective medical expert, about each respective topic; derived from logistic regression models that regress knowledge assessments on MFW and the controls mentioned in the text. Values closer to 1 on the primary (left-hand side) y-axis indicate an increased likelihood of believing that one knows more than experts. Please consult the Supplemental Materials for full model output.

Online Methods

Methods Table 1. Comparison of Sample Characteristics to National Benchmarks (Studies 1 and 2)

Variable	Study 1 (Raw)	Study 2 (Raw)	Study 2 (Weighted)	Benchmark	Benchmark Source
Female	51%	55%	52%	51%	CPS 2018
College Degree	40%	44%	35%	31%	CPS 2018
Black	12%	13%	14%	13%	CPS 2018
White	74%	70%	64%	62%	CPS 2018
Hispanic	13%	11%	17%	18%	CPS 2018
Democrat	39%	37%	40%	34%	ANES (Wgt.)
Republican	36%	29%	34%	28%	ANES (Wgt.)
Independent	22%	29%	28%	32%	ANES (Wgt.)
Mean Age	44	46	47	47	ANES (Wgt.)
Median Income	$30-34,999	$ 35 - 39,000	$ 55 - 59,999	$ 55-59,999	ANES (Wgt.)

Note. Comparison of our data to known population benchmarks. CPS = Current Population Survey (US Census, 2018). ANES = American National Election Study (2016). We prefer to rely on CPS given its sample size and representativeness, but make use of weighted ANES data whenever it was not possible to use CPS (e.g., CPS does not ask questions about Party ID). Weights in column two adjust for gender, education, race, age, and income. Party ID is **not included** in our weighting formula, and is shown only due to the potential interests of those who might use or otherwise consume this data. N (Study 1) = 509; N (Study 2) = 4,998.

Supplementary Figures

Figure S1. Replication of Figure 3 using Graded Response Modeling

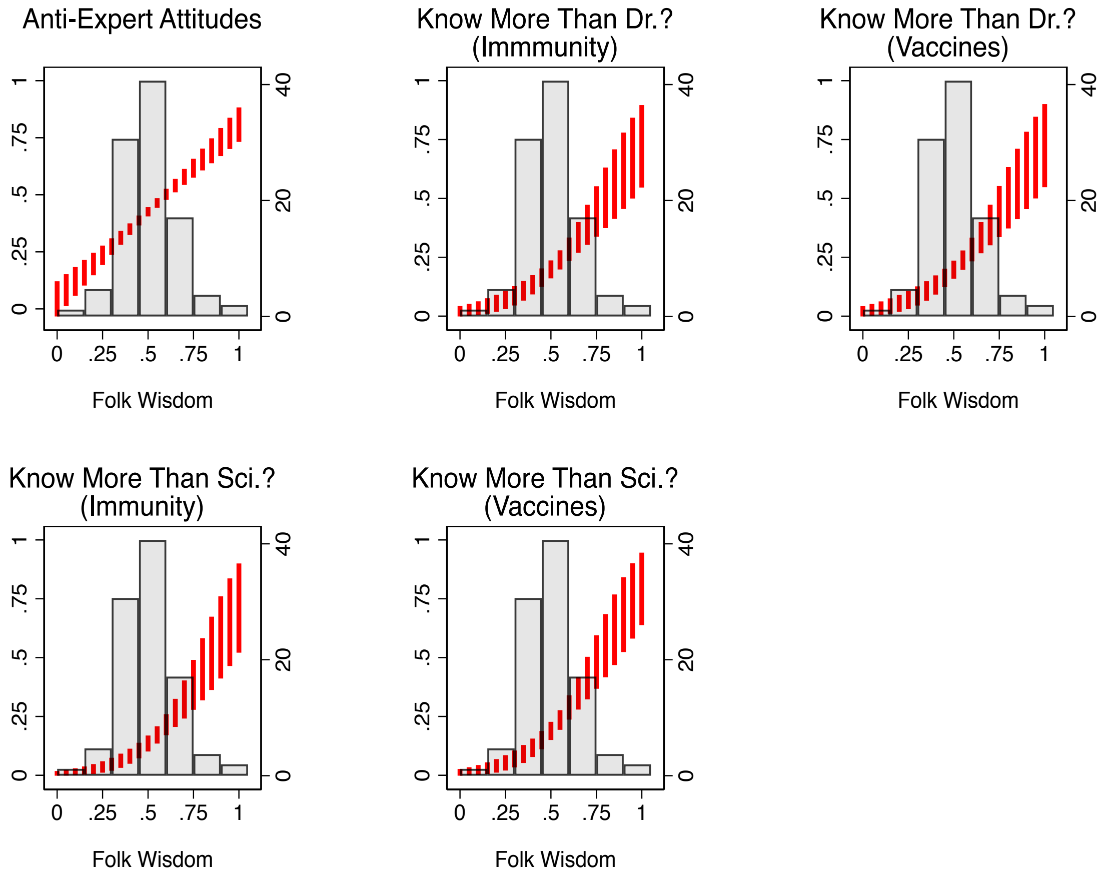

Note. This figure replicates the output presented in Figure 3 in the main text, swapping the MFW scale derived from the 2PL application of IRT for one derived via GRM. Please consult the note accompanying that figure, as all other information about this figure is unchanged.

Figure S2. Replication of Figure 4 using Graded Response Modeling

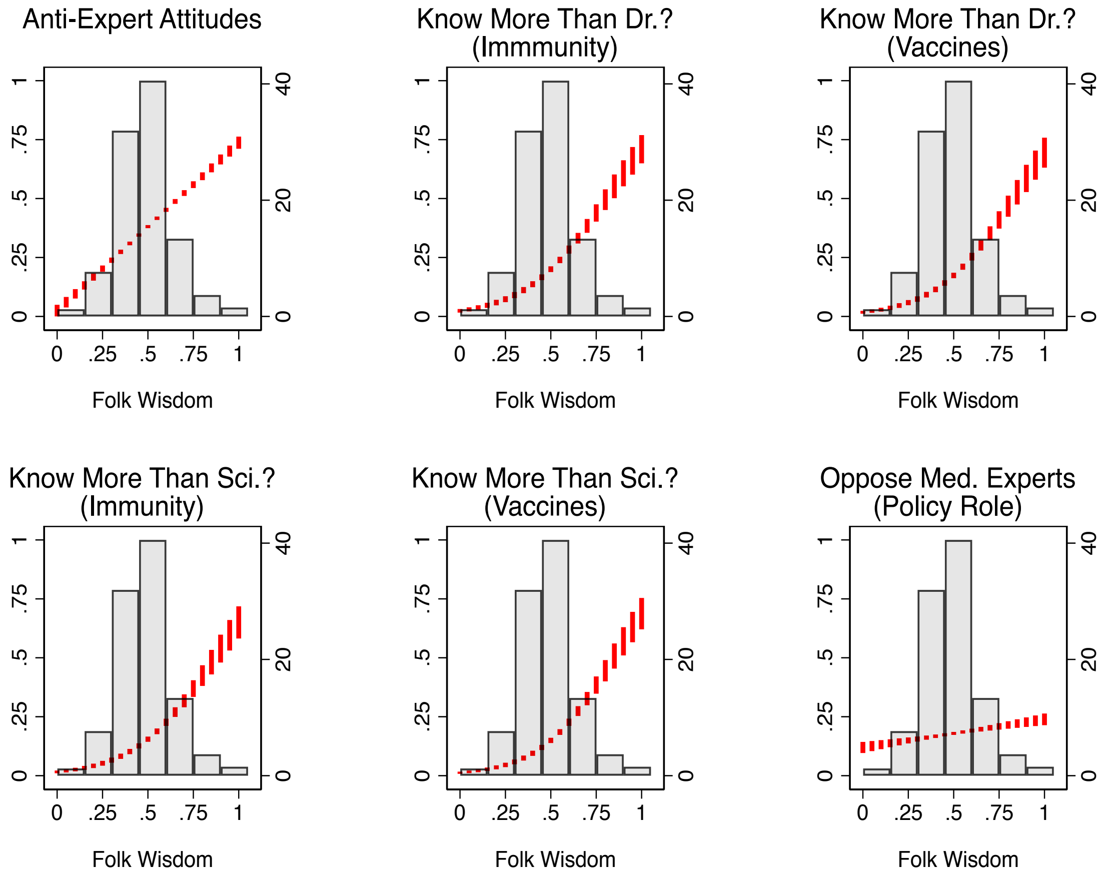

Note. This figure replicates the output presented in Figure 4 in the main text, swapping the MFW scale derived from the 2PL application of IRT for one derived via GRM. Please consult the note accompanying that figure, as all other information about this figure is unchanged.

Figure S3. Replication of Figure 3 using Short Form MFW Scale

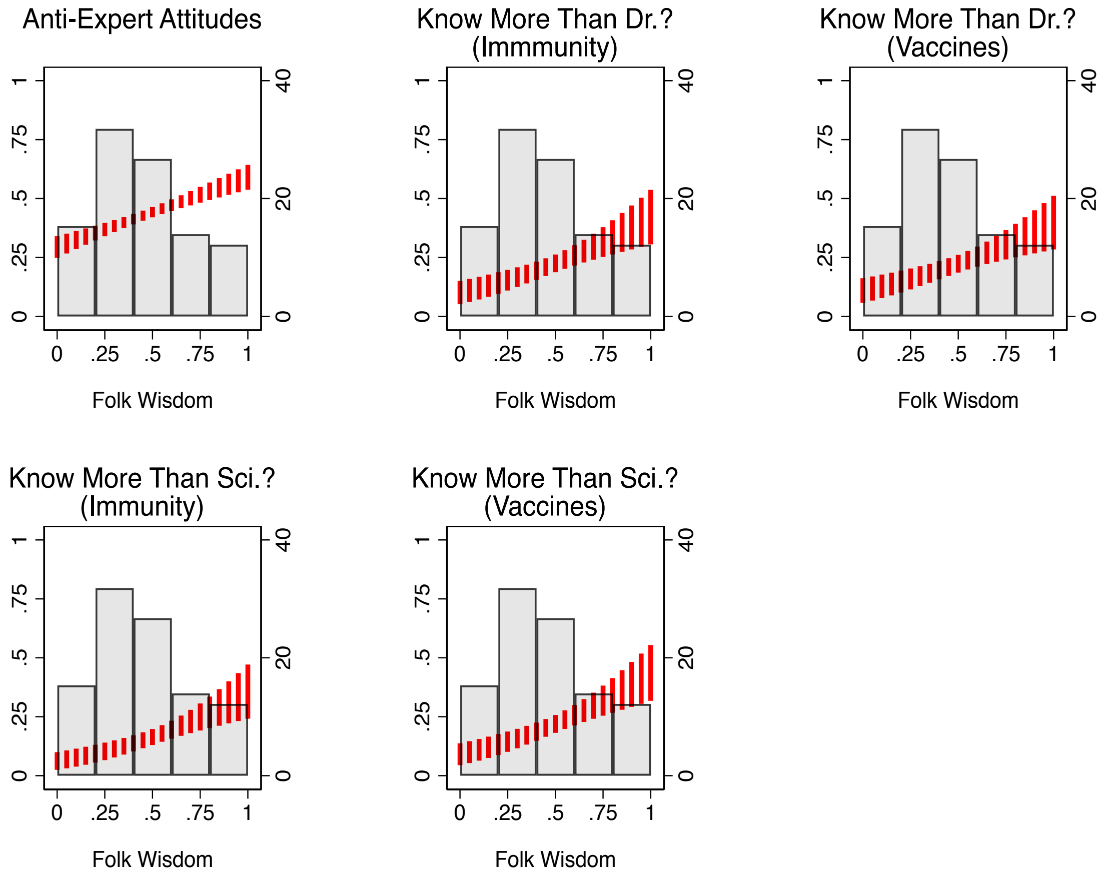

Note. This figure replicates the output presented in Figure 3 in the main text, swapping the MFW scale derived from the 2PL application of IRT for the short-form scale discussed in the main text. Please consult the note accompanying that figure, as all other information about this figure is unchanged.

Figure S4. Replication of Figure 4 using Short Form MFW Scale

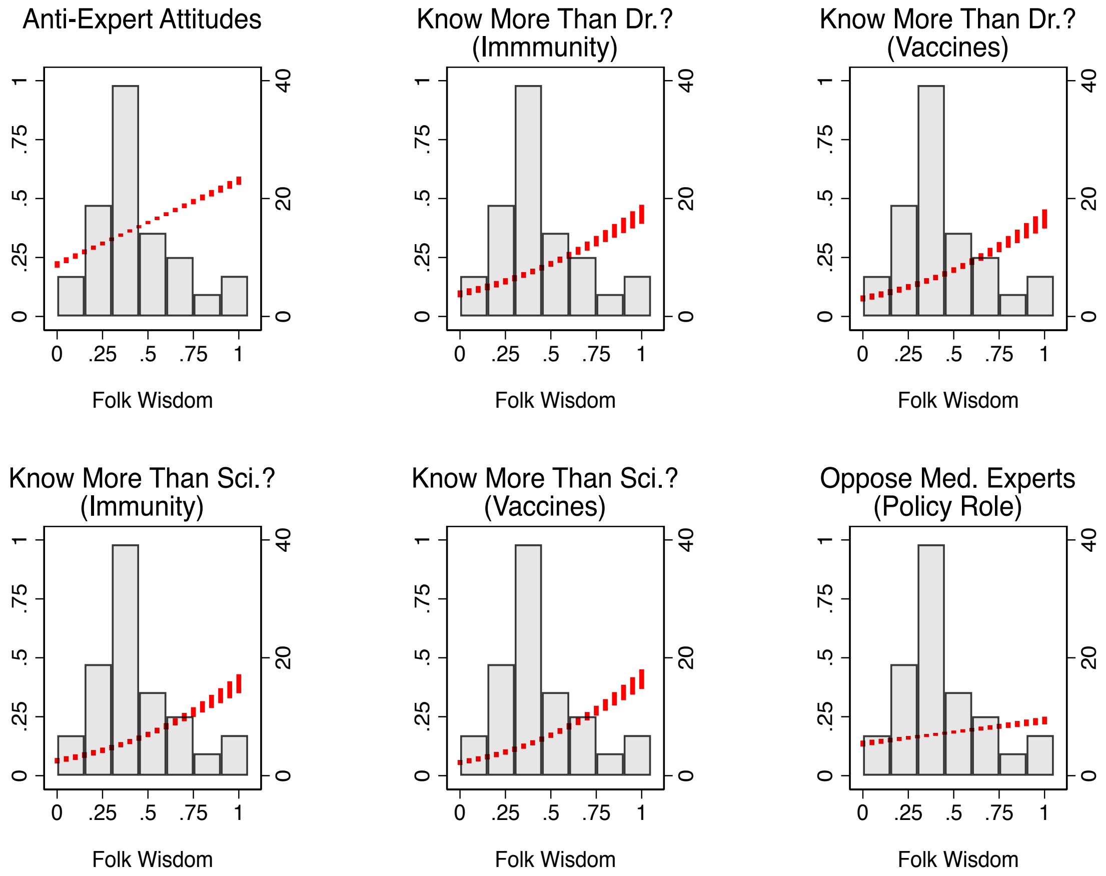

Note. This figure replicates the output presented in Figure 4 in the main text, swapping the MFW scale derived from the 2PL application of IRT for the short-form scale discussed in the main text. Please consult the note accompanying that figure, as all other information about this figure is unchanged.

Figure S5. Replication of Figure 2 with Post-stratification Weights

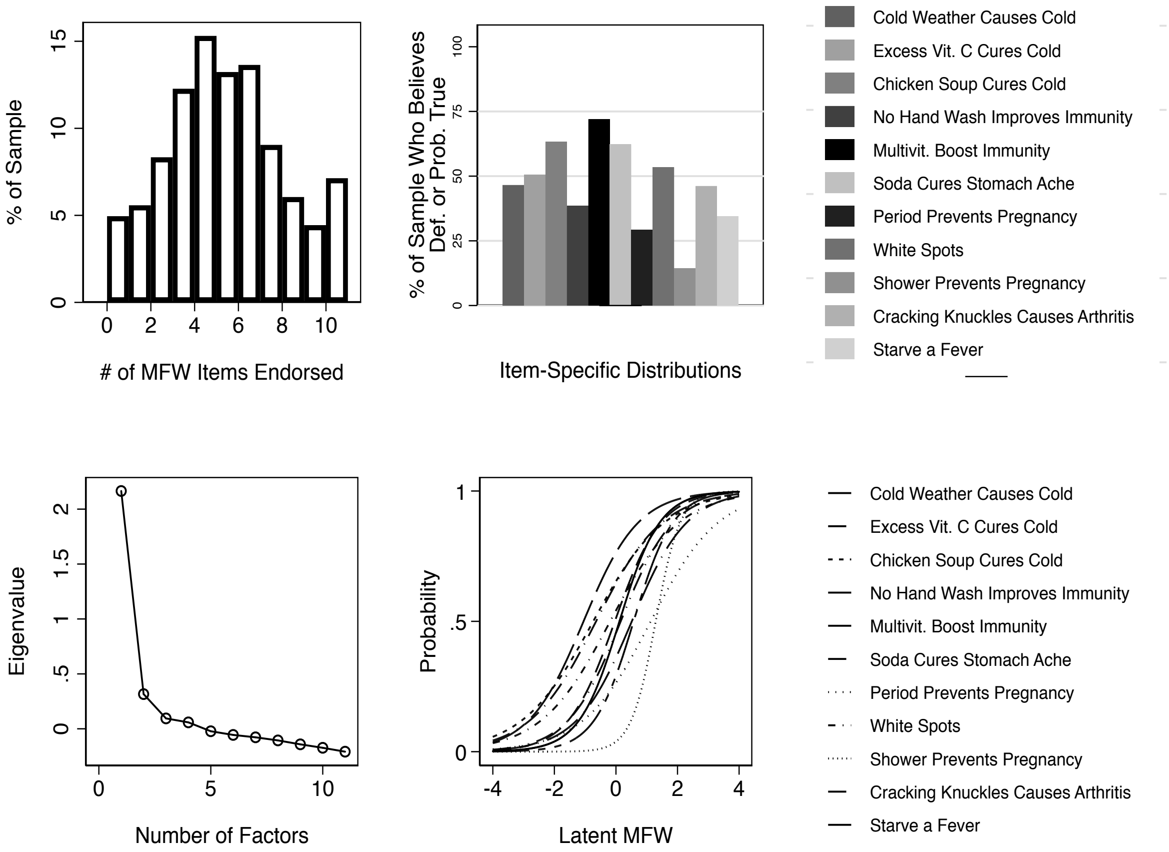

Note. This figure replicates the output presented in Figure 2 in the main text, applying survey weights to both the distribution of the count of folk theories endorsed, and the frequency of each specific theory endorsed. It also applies survey weights to the PCA procedure used to calculate the Eigenvalues summarized in the scree plot, as well as to the 2PL IRT procedure used to estimate item characteristic curves.

Supplementary Tables

Table S1. Correlates of the MFW Scale and Individual Items (Study 1)

	Full	Weather	Vitamin C	Soup	Nat. Imm.	Vitamins	Soda	Period	Spots	Shower	Knuckles	Starve
Sci. Know	-0.21*	-1.85*	-1.02*	-0.53	-1.41*	-0.73	-0.93*	-1.01*	-1.11*	-2.22*	-0.92*	-1.30*
	(0.04)	(0.41)	(0.38)	(0.40)	(0.40)	(0.42)	(0.40)	(0.42)	(0.39)	(0.60)	(0.38)	(0.42)
Consp. Ideation	0.22*	0.16	1.23*	1.25*	0.98*	0.16	1.17*	0.89	1.32*	1.70*	1.50*	2.28*
	(0.04)	(0.45)	(0.43)	(0.45)	(0.46)	(0.46)	(0.44)	(0.49)	(0.44)	(0.76)	(0.44)	(0.51)
Healthy	0.08*	0.87*	0.27	0.56*	0.24	0.27	0.41	0.62*	0.41	0.03	0.33	0.52
	(0.02)	(0.26)	(0.24)	(0.25)	(0.26)	(0.26)	(0.24)	(0.29)	(0.24)	(0.38)	(0.24)	(0.27)
Difficulty Seeing Dr.	0.07*	0.41	0.20	0.14	0.40	0.45	0.07	0.74*	0.27	0.75*	0.35	0.44
	(0.02)	(0.26)	(0.25)	(0.26)	(0.25)	(0.28)	(0.25)	(0.26)	(0.25)	(0.33)	(0.24)	(0.26)
HS	-0.01	0.73	0.37	0.06	-0.29	-0.13	0.18	-0.07	-0.15	-1.24	-0.31	0.28
	(0.06)	(0.70)	(0.61)	(0.66)	(0.62)	(0.71)	(0.62)	(0.67)	(0.66)	(0.76)	(0.62)	(0.65)
Some Coll.	-0.03	0.70	0.61	0.08	-0.94	0.07	0.19	-0.04	-0.60	-1.01	-0.32	-0.20
	(0.06)	(0.72)	(0.63)	(0.68)	(0.65)	(0.73)	(0.64)	(0.70)	(0.68)	(0.79)	(0.65)	(0.68)
Coll.	-0.04	0.98	0.20	0.24	-0.56	-0.24	-0.38	-0.32	-0.49	-1.19	-0.49	-0.11
	(0.06)	(0.71)	(0.62)	(0.67)	(0.63)	(0.72)	(0.63)	(0.69)	(0.68)	(0.79)	(0.64)	(0.67)
Age	-0.06	-1.58*	-0.09	0.89	-0.46	-0.52	0.04	0.58	-1.07*	-0.49	-0.52	-0.52
	(0.04)	(0.47)	(0.43)	(0.46)	(0.46)	(0.47)	(0.45)	(0.49)	(0.44)	(0.72)	(0.43)	(0.48)
Black	0.10*	1.07*	0.10	0.89*	0.40	0.30	0.16	0.24	0.24	1.04*	0.51	0.87*
	(0.03)	(0.37)	(0.32)	(0.39)	(0.31)	(0.37)	(0.33)	(0.34)	(0.33)	(0.38)	(0.32)	(0.32)
Hisp.	-0.03	0.27	0.36	-0.20	-0.42	-0.15	-0.50	0.05	-0.13	-1.13*	-0.31	0.01
	(0.03)	(0.31)	(0.31)	(0.30)	(0.32)	(0.33)	(0.30)	(0.33)	(0.30)	(0.50)	(0.29)	(0.31)
Income	-0.01	-1.20*	-0.24	-0.26	-0.48	0.12	0.95*	0.35	-0.12	-0.29	-0.06	0.29
	(0.04)	(0.42)	(0.40)	(0.41)	(0.42)	(0.43)	(0.42)	(0.45)	(0.40)	(0.65)	(0.40)	(0.44)
Female	-0.04*	-0.25	-0.30	0.12	-0.45*	-0.12	0.10	-0.64*	0.16	-0.75*	-0.04	-0.37
	(0.02)	(0.21)	(0.20)	(0.21)	(0.21)	(0.22)	(0.20)	(0.22)	(0.20)	(0.31)	(0.20)	(0.22)
Satisficing	0.14*	0.34	0.05	0.45	0.73*	0.21	0.37	1.93*	0.20	2.21*	0.06	0.89*
	(0.03)	(0.40)	(0.36)	(0.40)	(0.36)	(0.43)	(0.39)	(0.39)	(0.38)	(0.41)	(0.35)	(0.37)
β_0	0.46*	0.31	-0.18	-0.69	0.43	1.36	-0.13	-1.53	0.47	-0.58	-0.09	-1.62*
	(0.07)	(0.81)	(0.71)	(0.77)	(0.74)	(0.82)	(0.73)	(0.79)	(0.76)	(0.94)	(0.73)	(0.77)
N	481.00	479.00	481.00	480.00	481.00	479.00	480.00	479.00	481.00	478.00	481.00	479.00

* $p < 0.05$, *two-tailed*

Note. OLS parameter estimates (column 1) and logistic regression parameter estimates (columns 2-12) presented with standard errors in parentheses. Note that the full question wording for the items used to construct each outcome variable can be found in the first section of the Supplemental Materials. Note also that the coding and measurement of each item included as a covariate in these models is described at length in the Online Methods.

Table S2. Correlates of the MFW Scale and Individual Items (Study 2)

	Full	Weather	Vitamin C	Soup	Nat. Imm.	Vitamins	Soda	Period	Spots	Shower	Knuckles	Starve
Healthy	0.06*	0.28*	0.36*	0.33*	0.19*	0.31*	0.01	0.40*	0.16*	0.85*	0.18*	0.37*
	(0.01)	(0.08)	(0.08)	(0.08)	(0.08)	(0.08)	(0.08)	(0.09)	(0.08)	(0.16)	(0.08)	(0.09)
Difficulty Seeing Dr.	0.10*	0.49*	0.37*	0.28*	0.32*	0.21*	0.37*	0.52*	0.43*	1.25*	0.53*	0.62*
	(0.01)	(0.08)	(0.07)	(0.07)	(0.07)	(0.08)	(0.08)	(0.08)	(0.07)	(0.10)	(0.07)	(0.07)
HS	-0.01	-0.30	-0.06	0.08	-0.20	-0.06	-0.00	0.15	0.07	-0.38	-0.07	0.05
	(0.02)	(0.23)	(0.21)	(0.21)	(0.21)	(0.24)	(0.22)	(0.24)	(0.21)	(0.29)	(0.21)	(0.22)
Some Coll.	-0.05*	-0.51*	-0.27	0.00	-0.57*	-0.18	-0.04	0.07	-0.28	-0.52	-0.40	-0.15
	(0.02)	(0.23)	(0.21)	(0.22)	(0.21)	(0.24)	(0.22)	(0.25)	(0.21)	(0.30)	(0.21)	(0.22)
Coll.	-0.06*	-0.47*	-0.33	-0.11	-0.60*	-0.17	-0.24	0.21	-0.19	-0.42	-0.42*	-0.25
	(0.02)	(0.23)	(0.21)	(0.21)	(0.21)	(0.24)	(0.22)	(0.24)	(0.21)	(0.29)	(0.21)	(0.22)
Age	-0.19*	-2.78*	-0.98*	0.97*	-0.82*	-1.02*	-0.77*	-0.09	-1.20*	-2.53*	-0.76*	-0.59*
	(0.01)	(0.16)	(0.15)	(0.15)	(0.16)	(0.16)	(0.15)	(0.17)	(0.15)	(0.27)	(0.15)	(0.16)
Black	0.11*	1.02*	0.68*	0.57*	0.62*	0.35*	0.40*	0.35*	0.49*	0.60*	0.13	0.73*
	(0.01)	(0.10)	(0.10)	(0.10)	(0.09)	(0.11)	(0.10)	(0.10)	(0.10)	(0.12)	(0.09)	(0.09)
Hisp.	0.05*	0.48*	0.34*	0.29*	0.28*	0.21	0.16	0.21	0.14	0.22	0.03	0.43*
	(0.01)	(0.11)	(0.10)	(0.10)	(0.10)	(0.12)	(0.11)	(0.11)	(0.10)	(0.14)	(0.10)	(0.10)
Income	-0.01	-0.56*	0.14	-0.30*	0.07	-0.02	-0.12	0.25	0.02	0.69*	-0.17	-0.08
	(0.01)	(0.13)	(0.12)	(0.12)	(0.12)	(0.13)	(0.12)	(0.13)	(0.12)	(0.18)	(0.12)	(0.13)
Female	-0.05*	-0.54*	-0.14*	-0.05	-0.25*	-0.02	-0.05	-0.68*	0.08	-0.90*	-0.13*	-0.41*
	(0.01)	(0.07)	(0.06)	(0.06)	(0.06)	(0.07)	(0.06)	(0.07)	(0.06)	(0.10)	(0.06)	(0.07)
Satisficing	0.14*	0.12	0.45*	0.14	1.03*	-0.53*	0.07	1.10*	0.25	1.88*	0.40*	1.16*
	(0.02)	(0.19)	(0.18)	(0.18)	(0.18)	(0.18)	(0.19)	(0.18)	(0.18)	(0.19)	(0.18)	(0.19)
β_0	0.51*	1.22*	0.10	-0.13	-0.04	1.15*	0.80*	-1.36*	0.36	-1.86*	0.29	-0.76*
	(0.02)	(0.24)	(0.22)	(0.23)	(0.22)	(0.25)	(0.23)	(0.26)	(0.22)	(0.33)	(0.22)	(0.24)
N	4705.00	4703.00	4704.00	4703.00	4704.00	4705.00	4704.00	4703.00	4704.00	4705.00	4702.00	4702.00

* $p < 0.05$, *two-tailed*

Note. OLS parameter estimates (column 1) and logistic regression parameter estimates (columns 2-12) presented with standard errors in parentheses. Note that the full question wording for the items used to construct each outcome variable can be found in the first section of the Supplemental Materials. Note also that the coding and measurement of each item included as a covariate in these models is described at length in the Online Methods.

Table S3. The Effect of MFW on Health Behavior (Study 1)

	Stay Home	Wash Hands	Seatbelt	Doctor
MFW	0.83*	0.33	-0.56	1.54*
	(0.41)	(0.47)	(0.54)	(0.41)
Democrat	0.61*	0.37	0.50	0.73*
	(0.25)	(0.30)	(0.30)	(0.25)
Republican	0.34	0.10	0.93*	0.58*
	(0.25)	(0.30)	(0.33)	(0.25)
Individualism	-0.25	0.88	0.21	-0.94
	(0.52)	(0.63)	(0.79)	(0.53)
Healthy	-0.03	0.51*	0.36	-0.37
	(0.22)	(0.25)	(0.30)	(0.22)
Difficulty Seeing Dr.	0.33	0.34	-0.26	0.46*
	(0.21)	(0.26)	(0.27)	(0.21)
HS	-0.95	0.28	-0.42	0.21
	(0.60)	(0.65)	(0.69)	(0.55)
Some Coll.	-0.77	-0.13	-0.28	0.32
	(0.61)	(0.66)	(0.72)	(0.57)
Coll.	-1.29*	0.24	0.12	0.19
	(0.61)	(0.66)	(0.72)	(0.56)
Age	0.02	1.17*	1.38*	-0.36
	(0.39)	(0.47)	(0.58)	(0.39)
Black	-0.51	-0.69*	-0.26	0.09
	(0.27)	(0.30)	(0.34)	(0.28)
Hisp.	0.41	0.69*	0.29	0.17
	(0.27)	(0.32)	(0.35)	(0.26)
Income	0.46	0.34	0.46	0.45
	(0.35)	(0.42)	(0.52)	(0.35)
Female	0.55*	0.87*	0.08	0.54*
	(0.17)	(0.21)	(0.24)	(0.17)
Satisficing	-0.72*	-1.52*	-1.50*	0.24
	(0.30)	(0.34)	(0.35)	(0.30)
τ_1	-2.29*	-1.64	-2.98*	-0.99
	(0.77)	(0.90)	(1.00)	(0.73)
τ_2	-0.71	0.11	-1.66	1.11
	(0.77)	(0.87)	(0.97)	(0.73)
τ_3	0.81	1.57	-0.43	2.52*
	(0.77)	(0.87)	(0.96)	(0.73)
N	479.00	481.00	480.00	481.00

* $p < 0.05$, *two-tailed*

Note. Ordered logistic regression parameter estimates presented with standard errors in parentheses. Note that the full question wording for the items used to construct each outcome variable can be found in the first section of the Supplemental Materials. Note also that the coding and measurement of each item included as a covariate in these models is described at length in the Online Methods.

Table S4. The Effect of MFW on Health Behavior (Study 2)

	Stay Home	Wash Hands	Seatbelt	Doctor
MFW	1.05*	0.27	-0.58*	2.09*
	(0.13)	(0.16)	(0.18)	(0.14)
Individualism	0.23	-0.21	-0.02	1.06*
	(0.13)	(0.15)	(0.18)	(0.13)
Democrat	0.09	0.18*	0.19	0.32*
	(0.08)	(0.09)	(0.10)	(0.08)
Republican	0.13	0.08	0.27*	0.21*
	(0.08)	(0.09)	(0.11)	(0.08)
Healthy	-0.01	0.23*	0.23*	-0.23*
	(0.07)	(0.08)	(0.10)	(0.07)
Difficulty Seeing Dr.	0.26*	-0.31*	-0.57*	0.37*
	(0.07)	(0.07)	(0.08)	(0.07)
HS	0.48*	0.19	0.21	0.07
	(0.20)	(0.22)	(0.23)	(0.20)
Some Coll.	0.35	0.26	0.30	0.01
	(0.20)	(0.22)	(0.23)	(0.20)
Coll.	0.52*	0.18	0.29	0.12
	(0.20)	(0.22)	(0.23)	(0.20)
Age	0.66*	1.00*	1.60*	0.75*
	(0.14)	(0.16)	(0.21)	(0.14)
Black	0.22*	0.34*	-0.05	0.22*
	(0.09)	(0.10)	(0.11)	(0.09)
Hisp.	0.08	0.26*	-0.15	0.16
	(0.09)	(0.11)	(0.11)	(0.09)
Income	-0.04	0.26*	0.30*	0.15
	(0.10)	(0.12)	(0.15)	(0.11)
Female	0.34*	0.48*	0.37*	0.21*
	(0.06)	(0.06)	(0.08)	(0.06)
Satisficing	-0.13	-1.22*	-1.58*	0.18
	(0.15)	(0.16)	(0.16)	(0.15)
τ_1	-0.77*	-3.31*	-3.08*	0.08
	(0.23)	(0.28)	(0.28)	(0.23)
τ_2	0.93*	-1.22*	-1.72*	2.36*
	(0.23)	(0.25)	(0.27)	(0.23)
τ_3	2.56*	0.40	-0.49	3.69*
	(0.23)	(0.25)	(0.27)	(0.23)
N	4703.00	4702.00	4703.00	4703.00

* $p < 0.05$, *two-tailed*

Note. Ordered logistic regression parameter estimates presented with standard errors in parentheses. Note that the full question wording for the items used to construct each outcome variable can be found in the first section of the Supplemental Materials. Note also that the coding and measurement of each item included as a covariate in these models is described at length in the Online Methods.

Table S5. Full Output for Models Used to Build Figure 3

	Anti-Exp.	Dr. (Immunity)	Dr. (Vax)	Sci (Immunity)	Sci (Vax.)
MFW	0.41*	3.25*	3.07*	3.42*	3.65*
	(0.05)	(0.62)	(0.61)	(0.68)	(0.64)
Democrat	-0.05	1.01*	0.48	0.99*	0.80*
	(0.03)	(0.41)	(0.38)	(0.45)	(0.40)
Republican	0.02	1.05*	0.46	0.85	0.73
	(0.03)	(0.42)	(0.39)	(0.46)	(0.41)
Individualism	0.07	-1.21	-0.75	-1.01	0.19
	(0.07)	(0.81)	(0.81)	(0.91)	(0.83)
Difficulty Seeing Dr.	0.05	0.70*	0.88*	1.01*	0.69*
	(0.03)	(0.29)	(0.28)	(0.30)	(0.29)
HS	0.05	-0.27	0.06	0.18	0.74
	(0.07)	(0.77)	(0.80)	(0.90)	(0.91)
Some Coll.	0.07	-0.38	-0.14	0.29	0.86
	(0.07)	(0.80)	(0.83)	(0.93)	(0.93)
Coll.	0.07	0.18	0.50	0.84	1.45
	(0.07)	(0.79)	(0.81)	(0.91)	(0.92)
Age	0.06	0.74	0.67	-0.17	0.03
	(0.05)	(0.59)	(0.61)	(0.68)	(0.63)
Black	0.03	0.83*	0.48	0.51	0.40
	(0.04)	(0.36)	(0.37)	(0.39)	(0.37)
Hisp.	0.04	0.64	0.80*	0.62	0.91*
	(0.03)	(0.35)	(0.35)	(0.37)	(0.36)
Income	-0.13*	-0.47	0.07	-0.27	-0.58
	(0.04)	(0.51)	(0.50)	(0.55)	(0.52)
Female	0.03	-0.30	-0.05	-0.20	0.02
	(0.02)	(0.26)	(0.26)	(0.29)	(0.26)
Satisficing	0.16*	0.80*	0.80*	0.47	1.01*
	(0.04)	(0.39)	(0.39)	(0.41)	(0.40)
Sci. Know	-0.08	-0.28	-0.35	0.04	-0.15
	(0.04)	(0.51)	(0.51)	(0.57)	(0.52)
β_0	0.15	-3.43*	-3.65*	-4.51*	-5.33*
	(0.09)	(1.13)	(1.12)	(1.28)	(1.25)
N	482.00	479.00	470.00	480.00	469.00

* $p < 0.05$, *two-tailed*

Note. OLS parameter estimates (column 1) and logistic regression parameter estimates (columns 2-6) presented with standard errors in parentheses. Note that the full question wording for the items used to construct each outcome variable can be found in the first section of the Supplemental Materials. Note also that the coding and measurement of each item included as a covariate in these models is described at length in the Online Methods.

Table S6. Full Output for Models Used to Build Figure 4

	Anti-Exp.	Dr. (Immunity)	Dr. (Vax)	Sci (Immunity)	Sci (Vax.)	Policy Role
MFW	0.45*	3.14*	3.30*	3.36*	3.61*	0.10*
	(0.02)	(0.20)	(0.21)	(0.21)	(0.22)	(0.02)
Individualism	0.11*	1.16*	1.23*	1.48*	1.24*	-0.04*
	(0.02)	(0.19)	(0.20)	(0.21)	(0.21)	(0.02)
Democrat	-0.06*	0.27*	0.29*	0.39*	0.16	-0.05*
	(0.01)	(0.12)	(0.12)	(0.13)	(0.13)	(0.01)
Republican	0.02	0.36*	0.39*	0.45*	0.32*	-0.01
	(0.01)	(0.12)	(0.13)	(0.14)	(0.13)	(0.01)
Difficulty Seeing Dr.	0.06*	0.23*	0.42*	0.25*	0.47*	0.02*
	(0.01)	(0.09)	(0.09)	(0.10)	(0.09)	(0.01)
HS	-0.05*	-0.31	-0.38	-0.47	-0.21	0.03
	(0.02)	(0.26)	(0.26)	(0.27)	(0.28)	(0.02)
Some Coll.	-0.06*	-0.21	-0.31	-0.39	-0.05	0.01
	(0.02)	(0.26)	(0.27)	(0.28)	(0.29)	(0.02)
Coll.	-0.07*	-0.21	-0.32	-0.33	-0.05	-0.00
	(0.02)	(0.26)	(0.26)	(0.27)	(0.28)	(0.02)
Age	0.08*	-0.09	-0.30	0.08	-0.64*	-0.10*
	(0.02)	(0.20)	(0.22)	(0.23)	(0.23)	(0.02)
Black	0.04*	0.32*	0.34*	0.24*	0.15	0.02
	(0.01)	(0.11)	(0.11)	(0.12)	(0.12)	(0.01)
Hisp.	0.02	0.37*	0.24*	0.30*	0.31*	-0.01
	(0.01)	(0.12)	(0.12)	(0.13)	(0.13)	(0.01)
Income	0.00	0.35*	0.35*	0.51*	0.42*	-0.04*
	(0.01)	(0.15)	(0.16)	(0.16)	(0.16)	(0.01)
Female	0.02*	-0.13	-0.16	-0.40*	-0.34*	-0.01*
	(0.01)	(0.08)	(0.08)	(0.09)	(0.09)	(0.01)
Satisficing	0.15*	0.53*	0.32	0.65*	0.39*	0.17*
	(0.02)	(0.18)	(0.19)	(0.19)	(0.19)	(0.02)
β_0	0.12*	-3.68*	-3.84*	-4.21*	-4.16*	0.21*
	(0.03)	(0.30)	(0.31)	(0.32)	(0.33)	(0.03)
N	4703.00	4701.00	4694.00	4700.00	4692.00	4703.00

* $p < 0.05$, *two-tailed*

Note. OLS parameter estimates (columns 1 and 7) and logistic regression parameter estimates (columns 2-6) presented with standard errors in parentheses. Note that the full question wording for the items used to construct each outcome variable can be found in the first section of the Supplemental Materials. Note also that the coding and measurement of each item included as a covariate in these models is described at length in the Online Methods.

Table S7. Replication of Table S3 using Graded Response Modeling

	Stay Home	Wash Hands	Seatbelt	Doctor
MFW	1.51*	0.68	-0.28	2.70*
	(0.62)	(0.71)	(0.82)	(0.62)
Democrat	0.61*	0.37	0.50	0.73*
	(0.25)	(0.30)	(0.30)	(0.25)
Republican	0.33	0.09	0.92*	0.55*
	(0.25)	(0.30)	(0.33)	(0.25)
Individualism	-0.19	0.91	0.26	-0.83
	(0.52)	(0.63)	(0.79)	(0.53)
Healthy	-0.04	0.50*	0.32	-0.38
	(0.22)	(0.25)	(0.30)	(0.22)
Difficulty Seeing Dr.	0.32	0.33	-0.28	0.46*
	(0.21)	(0.25)	(0.27)	(0.21)
HS	-0.99	0.27	-0.41	0.16
	(0.60)	(0.65)	(0.69)	(0.55)
Some Coll.	-0.81	-0.14	-0.25	0.27
	(0.61)	(0.66)	(0.72)	(0.57)
Coll.	-1.36*	0.22	0.15	0.08
	(0.61)	(0.66)	(0.71)	(0.56)
Age	-0.01	1.16*	1.39*	-0.41
	(0.39)	(0.47)	(0.57)	(0.39)
Black	-0.54	-0.71*	-0.30	0.06
	(0.27)	(0.30)	(0.34)	(0.28)
Hisp.	0.37	0.67*	0.32	0.10
	(0.26)	(0.32)	(0.35)	(0.26)
Income	0.49	0.36	0.49	0.49
	(0.35)	(0.42)	(0.52)	(0.35)
Female	0.56*	0.88*	0.09	0.55*
	(0.17)	(0.21)	(0.24)	(0.17)
Satisficing	-0.75*	-1.54*	-1.56*	0.19
	(0.30)	(0.34)	(0.35)	(0.31)
τ_1	-1.96*	-1.47	-2.81*	-0.43
	(0.81)	(0.93)	(1.03)	(0.76)
τ_2	-0.38	0.29	-1.49	1.67*
	(0.80)	(0.91)	(1.01)	(0.76)
τ_3	1.15	1.74	-0.26	3.09*
	(0.80)	(0.91)	(1.00)	(0.77)
N	479.00	481.00	480.00	481.00

* $p < 0.05$, *two-tailed*

Note. Table replicates the results of Supplemental Table 4, summarized in the main text, swapping the measure of MFW derived from the 2PL application of IRT for one derived via GRM. Please refer to the original table for additional information, as all other information accompanying this table is the same.

Table S8. Replication of Table S4 using Graded Response Modeling

	Stay Home	Wash Hands	Seatbelt	Doctor
MFW	1.72*	0.91*	-0.24	3.27*
	(0.21)	(0.24)	(0.27)	(0.21)
Individualism	0.19	-0.30	-0.12	1.00*
	(0.13)	(0.15)	(0.18)	(0.13)
Democrat	0.10	0.18*	0.18	0.33*
	(0.08)	(0.09)	(0.10)	(0.08)
Republican	0.13	0.07	0.25*	0.21*
	(0.08)	(0.09)	(0.11)	(0.08)
Healthy	-0.01	0.22*	0.21*	-0.23*
	(0.07)	(0.08)	(0.10)	(0.07)
Difficulty Seeing Dr.	0.25*	-0.33*	-0.61*	0.38*
	(0.07)	(0.07)	(0.08)	(0.07)
HS	0.49*	0.21	0.22	0.09
	(0.20)	(0.22)	(0.23)	(0.20)
Some Coll.	0.36	0.28	0.32	0.04
	(0.20)	(0.22)	(0.23)	(0.20)
Coll.	0.53*	0.20	0.31	0.14
	(0.20)	(0.22)	(0.23)	(0.20)
Age	0.64*	1.04*	1.65*	0.71*
	(0.14)	(0.16)	(0.20)	(0.14)
Black	0.20*	0.31*	-0.09	0.20*
	(0.09)	(0.10)	(0.11)	(0.09)
Hisp.	0.08	0.25*	-0.17	0.16
	(0.09)	(0.11)	(0.11)	(0.09)
Income	-0.02	0.28*	0.31*	0.18
	(0.10)	(0.13)	(0.15)	(0.11)
Female	0.34*	0.49*	0.39*	0.21*
	(0.05)	(0.06)	(0.08)	(0.06)
Satisficing	-0.13	-1.26*	-1.63*	0.21
	(0.15)	(0.16)	(0.16)	(0.15)
τ_1	-0.43	-3.03*	-2.97*	0.68*
	(0.24)	(0.29)	(0.30)	(0.24)
τ_2	1.27*	-0.93*	-1.60*	2.96*
	(0.24)	(0.26)	(0.29)	(0.24)
τ_3	2.90*	0.69*	-0.37	4.30*
	(0.24)	(0.26)	(0.28)	(0.25)
N	4703.00	4702.00	4703.00	4703.00

* $p < 0.05$, *two-tailed*

Note. Table replicates the results of Supplemental Table 4, summarized in the main text, swapping the measure of MFW derived from the 2PL application of IRT for one derived via GRM. Please refer to the original table for additional information, as all other information accompanying this table is the same.

Table S9. Replication of Table S3 using Short Form MFW Scale

	Stay Home	Wash Hands	Seatbelt	Doctor
MFW	0.63	-0.11	-0.57	1.30*
	(0.35)	(0.41)	(0.46)	(0.35)
Democrat	0.60*	0.36	0.51	0.69*
	(0.25)	(0.30)	(0.30)	(0.25)
Republican	0.36	0.11	0.92*	0.61*
	(0.25)	(0.30)	(0.33)	(0.25)
Individualism	-0.27	0.84	0.19	-0.95
	(0.52)	(0.63)	(0.79)	(0.53)
Healthy	-0.01	0.55*	0.36	-0.35
	(0.22)	(0.25)	(0.30)	(0.22)
Difficulty Seeing Dr.	0.33	0.38	-0.25	0.47*
	(0.21)	(0.26)	(0.27)	(0.21)
HS	-0.92	0.28	-0.42	0.31
	(0.60)	(0.65)	(0.69)	(0.55)
Some Coll.	-0.77	-0.14	-0.28	0.41
	(0.62)	(0.66)	(0.72)	(0.57)
Coll.	-1.28*	0.22	0.12	0.26
	(0.61)	(0.66)	(0.71)	(0.56)
Age	0.00	1.14*	1.39*	-0.42
	(0.39)	(0.47)	(0.58)	(0.39)
Black	-0.50	-0.64*	-0.24	0.11
	(0.27)	(0.30)	(0.34)	(0.28)
Hisp.	0.41	0.67*	0.27	0.20
	(0.27)	(0.32)	(0.35)	(0.26)
Income	0.43	0.30	0.45	0.42
	(0.35)	(0.42)	(0.52)	(0.35)
Female	0.54*	0.85*	0.07	0.53*
	(0.17)	(0.21)	(0.24)	(0.17)
Satisficing	-0.71*	-1.44*	-1.47*	0.21
	(0.31)	(0.34)	(0.35)	(0.31)
τ_1	-2.38*	-1.85*	-2.98*	-1.09
	(0.77)	(0.89)	(0.98)	(0.73)
τ_2	-0.81	-0.10	-1.66	1.01
	(0.76)	(0.86)	(0.95)	(0.72)
τ_3	0.71	1.35	-0.43	2.41*
	(0.76)	(0.86)	(0.95)	(0.73)
N	479.00	481.00	480.00	481.00

Note. Table replicates the results of Supplemental Table 4, swapping the measure of MFW derived from the 2PL application of IRT for the short form scale discussed in the main text. Please refer to the original table for additional information, as all other information accompanying this table is the same.

Table S10. Replication of Table S4 using Short Form MFW Scale

	Stay Home	Wash Hands	Seatbelt	Doctor
MFW	0.78*	0.16	-0.63*	1.57*
	(0.11)	(0.13)	(0.15)	(0.12)
Individualism	0.29*	-0.19	-0.00	1.17*
	(0.13)	(0.15)	(0.18)	(0.13)
Democrat	0.09	0.18*	0.19	0.32*
	(0.08)	(0.09)	(0.10)	(0.08)
Republican	0.14	0.08	0.27*	0.22*
	(0.08)	(0.09)	(0.11)	(0.08)
Healthy	-0.01	0.24*	0.24*	-0.23*
	(0.07)	(0.08)	(0.10)	(0.07)
Difficulty Seeing Dr.	0.27*	-0.30*	-0.56*	0.39*
	(0.07)	(0.07)	(0.08)	(0.07)
HS	0.46*	0.19	0.21	0.04
	(0.20)	(0.22)	(0.23)	(0.20)
Some Coll.	0.32	0.25	0.30	-0.04
	(0.20)	(0.22)	(0.23)	(0.20)
Coll.	0.48*	0.17	0.29	0.06
	(0.20)	(0.22)	(0.23)	(0.20)
Age	0.58*	0.98*	1.62*	0.58*
	(0.14)	(0.16)	(0.20)	(0.14)
Black	0.25*	0.35*	-0.04	0.27*
	(0.09)	(0.10)	(0.11)	(0.09)
Hisp.	0.09	0.27*	-0.15	0.19*
	(0.09)	(0.11)	(0.11)	(0.09)
Income	-0.06	0.26*	0.32*	0.12
	(0.10)	(0.12)	(0.15)	(0.11)
Female	0.33*	0.47*	0.37*	0.19*
	(0.05)	(0.06)	(0.08)	(0.06)
Satisficing	-0.14	-1.22*	-1.54*	0.17
	(0.16)	(0.16)	(0.16)	(0.16)
τ_1	-0.95*	-3.38*	-3.05*	-0.29
	(0.23)	(0.27)	(0.28)	(0.22)
τ_2	0.75*	-1.28*	-1.69*	1.98*
	(0.23)	(0.25)	(0.27)	(0.22)
τ_3	2.37*	0.34	-0.45	3.30*
	(0.23)	(0.24)	(0.26)	(0.23)
N	4703.00	4702.00	4703.00	4703.00

Note. Table replicates the results of Supplemental Table 4, swapping the measure of MFW derived from the 2PL application of IRT for the short form scale discussed in the main text. Please refer to the original table for additional information, as all other information accompanying this table is the same.

Table S11. IRT Parameters for MFW Scale: 2pl Application (Study 1)

Two-parameter logistic model — Number of obs = 497
Log likelihood = -3135.6105

	Coef.	Std. Err.	z	P>|z|	[95% Conf.	Interval]
**Cold**						
Discrim	1.1522	.1658398	6.95	0.000	.8271603	1.47724
Diff	-.0414848	.0985979	-0.42	0.674	-.2347332	.1517635
**Vitamin C**						
Discrim	1.316943	.1869144	7.05	0.000	.9505975	1.683289
Diff	-.3240951	.0949616	-3.41	0.001	-.5102164	-.1379739
**Soup**						
Discrim	1.188749	.1805298	6.58	0.000	.8349171	1.542581
Diff	-.7995803	.1279929	-6.25	0.000	-1.050442	-.5487187
**Nat. Immunity**						
Discrim	1.305553	.180562	7.23	0.000	.9516578	1.659448
Diff	.4374714	.1013791	4.32	0.000	.238772	.6361707
**Multivitamins**						
Discrim	.9160926	.1599649	5.73	0.000	.6025672	1.229618
Diff	-1.255849	.2070566	-6.07	0.000	-1.661673	-.8500257
**Soda**						
Discrim	1.249611	.1837945	6.80	0.000	.8893799	1.609841
Diff	-.6100602	.1110853	-5.49	0.000	-.8277834	-.392337
**Period**						
Discrim	.9768483	.1525264	6.40	0.000	.677902	1.275795
Diff	.9871142	.1646142	6.00	0.000	.6644763	1.309752
**White Spots**						
Discrim	1.185512	.1731511	6.85	0.000	.8461424	1.524882
Diff	-.2828692	.1002213	-2.82	0.005	-.4792994	-.0864391
**Showering**						
Discrim	2.160504	.3303847	6.54	0.000	1.512962	2.808046
Diff	1.329549	.1257941	10.57	0.000	1.082998	1.576101
**Knuckles**						
Discrim	1.270014	.1776769	7.15	0.000	.9217739	1.618254
Diff	.0016113	.092623	0.02	0.986	-.1799264	.1831491
**Starve a Fever**						
Discrim	2.161651	.3138057	6.89	0.000	1.546603	2.776699
Diff	.4137982	.0787285	5.26	0.000	.2594933	.5681032

Note. 2PL IRT parameters displayed for each of the eleven folk theories. Full question wording can be found in the Question Wording section in the Supplementary Materials (see word/phrase summarizes above each set of parameters for guidance). “Discrim” denotes the discrimination parameter (a) and “Diff” denotes difficulty (b).

Table S12. IRT Parameters for MFW Scale: 2pl Application (Study 2)

Two-parameter logistic model — Number of obs = 4,889
Log likelihood = -31395.896

	Coef.	Std. Err.	z	P>|z|	[95% Conf.	Interval]
**Cold**						
Discrim	1.511441	.0688847	21.94	0.000	1.376429	1.646453
Diff	.0953851	.0269822	3.54	0.000	.0425011	.1482692
**Vitamin C**						
Discrim	1.311522	.0607901	21.57	0.000	1.192375	1.430668
Diff	.0020231	.0289758	0.07	0.944	-.0547683	.0588146
**Soup**						
Discrim	.8720972	.0482917	18.06	0.000	.7774473	.9667471
Diff	-.6918255	.0485132	-14.26	0.000	-.7869096	-.5967414
**Nat. Immunity**						
Discrim	1.137123	.0539096	21.09	0.000	1.031462	1.242784
Diff	.561252	.0375668	14.94	0.000	.4876224	.6348817
**Multivitamins**						
Discrim	1.009033	.0551307	18.30	0.000	.9009787	1.117087
Diff	-1.126672	.0576137	-19.56	0.000	-1.239592	-1.013751
**Soda**						
Discrim	.9365631	.0497983	18.81	0.000	.8389604	1.034166
Diff	-.6358505	.0443961	-14.32	0.000	-.7228652	-.5488358
**Period**						
Discrim	1.016138	.0513264	19.80	0.000	.9155398	1.116736
Diff	1.076794	.0543907	19.80	0.000	.9701906	1.183398
**White Spots**						
Discrim	.8595899	.0461513	18.63	0.000	.769135	.9500448
Diff	-.2063215	.0394235	-5.23	0.000	-.2835901	-.1290528
**Showering**						
Discrim	2.366887	.1269975	18.64	0.000	2.117976	2.615797
Diff	1.364168	.0405644	33.63	0.000	1.284663	1.443672
**Knuckles**						
Discrim	1.120933	.0535601	20.93	0.000	1.015957	1.225909
Diff	.1248043	.0323855	3.85	0.000	.0613299	.1882788
**Starve a Fever**						
Discrim	1.520156	.0690919	22.00	0.000	1.384738	1.655573
Diff	.6273059	.0325821	19.25	0.000	.5634461	.6911657

Note. 2PL IRT parameters displayed for each of the eleven folk theories. Full question wording can be found in the Question Wording section in the Supplementary Materials (see word/phrase summarizes above each set of parameters for guidance). “Discrim” denotes the discrimination parameter (a) and “Diff” denotes difficulty (b).

Table S13. IRT Parameters for MFW Scale: GRM Application (Study 1)

Graded response model — Number of obs = 497

Log likelihood = -6276.5542

	Coef.	Std. Err.	z	P>|z|	[95% Conf.	Interval]
Cold Weather						
Discrim	1.196556	.1320583	9.06	0.000	.9377265	1.455385
Diff						
>=2	-1.384353	.1588564	-8.71	0.000	-1.695706	-1.073
>=3	-.0345431	.0942169	-0.37	0.714	-.2192048	.1501187
=4	1.396804	.1627346	8.58	0.000	1.07785	1.715758
Vitamin C						
Discrim	1.423839	.1463846	9.73	0.000	1.13693	1.710748
Diff						
>=2	-1.814278	.1747437	-10.38	0.000	-2.156769	-1.471787
>=3	-.301732	.0874618	-3.45	0.001	-.473154	-.1303099
=4	1.32553	.1402032	9.45	0.000	1.050737	1.600323
Soup						
Discrim	1.48523	.1540013	9.64	0.000	1.183393	1.787067
Diff						
>=2	-2.415359	.2289855	-10.55	0.000	-2.864163	-1.966556
>=3	-.6897486	.0972134	-7.10	0.000	-.8802833	-.4992139
=4	1.237258	.1328965	9.31	0.000	.9767861	1.497731
Nat. Immunity						
Discrim	1.098311	.1279921	8.58	0.000	.8474509	1.349171
Diff						
>=2	-.6726147	.1189981	-5.65	0.000	-.9058468	-.4393826
>=3	.4468493	.1105694	4.04	0.000	.2301374	.6635613
=4	1.557243	.186937	8.33	0.000	1.190853	1.923633
Multivitamins						
Discrim	1.116189	.1316649	8.48	0.000	.8581311	1.374248
Diff						
>=2	-3.097888	.3509521	-8.83	0.000	-3.785741	-2.410035
>=3	-1.082199	.1422053	-7.61	0.000	-1.360917	-.803482
=4	1.217023	.1601409	7.60	0.000	.9031522	1.530893
Soda						
Discrim	1.268636	.1350952	9.39	0.000	1.003854	1.533417
Diff						
>=2	-2.21747	.2228934	-9.95	0.000	-2.654333	-1.780607
>=3	-.61032	.1026549	-5.95	0.000	-.81152	-.4091201
=4	1.630362	.1724993	9.45	0.000	1.292269	1.968454
Period						
Discrim	.910353	.1174825	7.75	0.000	.6800916	1.140614
Diff						
>=2	-.6063785	.1340457	-4.52	0.000	-.8691033	-.3436537
>=3	1.013971	.1621598	6.25	0.000	.6961441	1.331799
=4	2.588903	.3213349	8.06	0.000	1.959098	3.218708
White Spots						
Discrim	1.288434	.1355724	9.50	0.000	1.022717	1.554151
Diff						
>=2	-2.384533	.2367284	-10.07	0.000	-2.848512	-1.920554
>=3	-.2993241	.092539	-3.23	0.001	-.4806973	-.117951

	Coef.	Std. Err.	z	P>|z|	[95% Conf.	Interval]
=4	1.800366	.1827507	9.85	0.000	1.442182	2.158551
**Showering**						
Discrim	1.467031	.1705892	8.60	0.000	1.132682	1.80138
Diff						
>=2	.5892034	.0970481	6.07	0.000	.3989926	.7794141
>=3	1.514123	.1592953	9.51	0.000	1.20191	1.826336
=4	2.365168	.2351149	10.06	0.000	1.904351	2.825985
**Knuckles**						
Discrim	1.330236	.1387822	9.59	0.000	1.058228	1.602244
Diff						
>=2	-1.368626	.1456541	-9.40	0.000	-1.654102	-1.083149
>=3	-.0253022	.0882686	-0.29	0.774	-.1983055	.1477011
=4	1.639455	.1671857	9.81	0.000	1.311777	1.967133
**Starve a Fever**						
Discrim	1.588662	.1567147	10.14	0.000	1.281506	1.895817
Diff						
>=2	-1.216869	.1206015	-10.09	0.000	-1.453243	-.9804943
>=3	.4361668	.0879368	4.96	0.000	.2638139	.6085197
=4	2.025608	.1792379	11.30	0.000	1.674308	2.376908

Note. GRM IRT parameters displayed for each of the eleven folk theories, used to create the MFW scales presented in supplementary analyses. Full question wording can be found in the Question Wording section in the Supplementary Materials (see word/phrase summarizes above each set of parameters for guidance). "Discrim" denotes the discrimination parameter (a) and "Diff" denotes difficulty (b).

Table S14. IRT Parameters for MFW Scale: GRM Application (Study 2)

Graded response model   Number of obs   =   4,889
Log likelihood = -62738.275

	Coef.	Std. Err.	z	P>|z|	[95% Conf.	Interval]
Cold Weather						
Discrim	1.417866	.0482697	29.37	0.000	1.323259	1.512473
Diff						
>=2	-1.023939	.038192	-26.81	0.000	-1.098794	-.9490844
>=3	.1019845	.0273899	3.72	0.000	.0483014	.1556677
=4	1.291535	.044452	29.05	0.000	1.204411	1.378659
Vitamin C						
Discrim	1.368703	.0462213	29.61	0.000	1.278111	1.459295
Diff						
>=2	-1.670095	.05321	-31.39	0.000	-1.774385	-1.565806
>=3	-.011585	.0277054	-0.42	0.676	-.0658866	.0427165
=4	1.547368	.050909	30.39	0.000	1.447588	1.647148
Soup						
Discrim	1.059788	.0402358	26.34	0.000	.980927	1.138649
Diff						
>=2	-2.655709	.0968501	-27.42	0.000	-2.845532	-2.465887
>=3	-.5899039	.0373677	-15.79	0.000	-.6631432	-.5166647
=4	1.715098	.064865	26.44	0.000	1.587965	1.842231
Nat. Immunity						
Discrim	.9228964	.0381433	24.20	0.000	.848137	.9976558
Diff						
>=2	-.6396094	.0426505	-15.00	0.000	-.7232028	-.556016
>=3	.6180953	.0427816	14.45	0.000	.534245	.7019456
=4	1.78027	.0743391	23.95	0.000	1.634568	1.925972
Multivitamins						
Discrim	1.064041	.0412455	25.80	0.000	.9832012	1.144881
Diff						
>=2	-2.887988	.106549	-27.10	0.000	-3.096821	-2.679156
>=3	-1.055678	.046906	-22.51	0.000	-1.147612	-.9637445
=4	1.406297	.0570412	24.65	0.000	1.294499	1.518096
Soda						
Discrim	1.069374	.0403	26.54	0.000	.9903875	1.14836
Diff						
>=2	-2.345476	.0846471	-27.71	0.000	-2.511381	-2.179571
>=3	-.573838	.0366957	-15.64	0.000	-.6457603	-.5019157
=4	1.725399	.0648998	26.59	0.000	1.598198	1.852601
Period						
Discrim	.9179125	.0387163	23.71	0.000	.8420299	.9937951
Diff						
>=2	-.3023277	.038108	-7.93	0.000	-.3770181	-.2276374
>=3	1.130616	.0549294	20.58	0.000	1.022957	1.238276
=4	2.614547	.1046785	24.98	0.000	2.409381	2.819713
White Spots						
Discrim	1.05388	.0398461	26.45	0.000	.9757827	1.131977
Diff						
>=2	-2.585673	.0938351	-27.56	0.000	-2.769587	-2.40176
>=3	-.200777	.0332011	-6.05	0.000	-.26585	-.135704

=4	2.248122	.0811116	27.72	0.000	2.089146	2.407098
Showering						
Discrim	1.64764	.0618047	26.66	0.000	1.526505	1.768775
Diff						
>=2	.6827838	.0301889	22.62	0.000	.6236146	.7419529
>=3	1.538289	.0479043	32.11	0.000	1.444398	1.63218
=4	2.425271	.0725811	33.41	0.000	2.283015	2.567528
Knuckles						
Discrim	1.131848	.0408408	27.71	0.000	1.051801	1.211894
Diff						
>=2	-1.362876	.0519643	-26.23	0.000	-1.464724	-1.261028
>=3	.1180171	.0314731	3.75	0.000	.0563309	.1797032
=4	1.902627	.0666993	28.53	0.000	1.771899	2.033356
Starve a Fever						
Discrim	1.443067	.0476462	30.29	0.000	1.349682	1.536452
Diff						
>=2	-1.098199	.0387204	-28.36	0.000	-1.174089	-1.022308
>=3	.5990459	.0312716	19.16	0.000	.5377546	.6603371
=4	2.08468	.0620868	33.58	0.000	1.962992	2.206368

Note. GRM IRT parameters displayed for each of the eleven folk theories, used to create the MFW scales presented in supplementary analyses. Full question wording can be found in the Question Wording section in the Supplementary Materials (see word/phrase summarizes above each set of parameters for guidance). “Discrim” denotes the discrimination parameter (a) and “Diff” denotes difficulty (b).

Table S15. IRT Parameters for Short Form MFW Scale (Study 1)

Two-parameter logistic model Number of obs = 497

Log likelihood = -1384.9539

	Coef.	Std. Err.	z	P>|z|	[95% Conf.	Interval]
Soup						
Discrim	1.161991	.2186072	5.32	0.000	.7335285	1.590453
Diff	-.8097623	.1430857	-5.66	0.000	-1.090205	-.5293196
Multivitamins						
Discrim	.7871686	.1747699	4.50	0.000	.4446258	1.129711
Diff	-1.409087	.2864231	-4.92	0.000	-1.970466	-.8477076
Showering						
Discrim	2.625815	.6262948	4.19	0.000	1.3983	3.85333
Diff	1.250072	.1260466	9.92	0.000	1.003026	1.497119
White Spots						
Discrim	1.251429	.2301787	5.44	0.000	.800287	1.702571
Diff	-.2673371	.0988435	-2.70	0.007	-.4610668	-.0736074
Starve a Fever						
Discrim	1.688251	.3119144	5.41	0.000	1.07691	2.299592
Diff	.4854536	.0936278	5.18	0.000	.3019465	.6689607

Note. Short form scale IRT parameters displayed for each of the eleven folk theories, used to create the MFW scales presented in supplementary analyses. Full question wording can be found in the Question Wording section in the Supplementary Materials (see word/phrase summarizes above each set of parameters for guidance). "Discrim" denotes the discrimination parameter (a) and "Diff" denotes difficulty (b).

Table S16. IRT Parameters for Short Form MFW Scale (Study 2)

```
Two-parameter logistic model                    Number of obs     =      4,889
Log likelihood = -13906.865
------------------------------------------------------------------------------
             |      Coef.   Std. Err.      z    P>|z|     [95% Conf. Interval]
-------------+----------------------------------------------------------------
Soup
     Discrim |   .8772173   .0600553    14.61   0.000      .759511    .9949236
        Diff |  -.6855226    .052634   -13.02   0.000    -.7886834   -.5823618
-------------+----------------------------------------------------------------
Multivitamins
     Discrim |   .7612292   .0591653    12.87   0.000     .6452673    .8771911
        Diff |  -1.392508   .0985314   -14.13   0.000    -1.585626    -1.19939
-------------+----------------------------------------------------------------
Showering
     Discrim |   2.297521   .1844298    12.46   0.000     1.936045    2.658997
        Diff |   1.387319   .0503254    27.57   0.000     1.288683    1.485955
-------------+----------------------------------------------------------------
White Spots
     Discrim |   .8111554   .0559088    14.51   0.000     .7015761    .9207347
        Diff |  -.2101183   .0419607    -5.01   0.000    -.2923598   -.1278768
-------------+----------------------------------------------------------------
Starve a Fever
     Discrim |   1.801098    .127029    14.18   0.000     1.552126     2.05007
        Diff |   .5905648   .0320972    18.40   0.000     .5276556    .6534741
------------------------------------------------------------------------------
```

Note. Short form scale IRT parameters displayed for each of the eleven folk theories, used to create the MFW scales presented in supplementary analyses. Full question wording can be found in the Question Wording section in the Supplementary Materials (see word/phrase summarizes above each set of parameters for guidance). "Discrim" denotes the discrimination parameter (a) and "Diff" denotes difficulty (b).

Table S17. Models Used to Produce Figure S1

	Anti-Exp.	Dr. (Immunity)	Dr. (Vax)	Sci (Immunity)	Sci (Vax.)
MFW	0.76*	5.15*	5.12*	6.06*	6.07*
	(0.07)	(0.98)	(0.97)	(1.07)	(1.04)
Democrat	-0.05	0.92*	0.41	0.91*	0.73
	(0.03)	(0.40)	(0.38)	(0.45)	(0.40)
Republican	0.00	0.90*	0.32	0.68	0.57
	(0.03)	(0.41)	(0.39)	(0.47)	(0.41)
Individualism	0.10	-1.08	-0.64	-0.78	0.33
	(0.06)	(0.81)	(0.82)	(0.93)	(0.84)
Difficulty Seeing Dr.	0.04	0.69*	0.85*	0.96*	0.64*
	(0.03)	(0.29)	(0.28)	(0.31)	(0.29)
HS	0.04	-0.40	-0.10	-0.08	0.51
	(0.07)	(0.74)	(0.78)	(0.88)	(0.90)
Some Coll.	0.06	-0.56	-0.36	-0.02	0.57
	(0.07)	(0.77)	(0.82)	(0.91)	(0.92)
Coll.	0.04	-0.13	0.15	0.38	1.01
	(0.07)	(0.76)	(0.80)	(0.89)	(0.91)
Age	0.05	0.65	0.59	-0.31	-0.08
	(0.05)	(0.59)	(0.61)	(0.70)	(0.64)
Black	0.01	0.83*	0.46	0.43	0.36
	(0.03)	(0.36)	(0.38)	(0.40)	(0.38)
Hisp.	0.02	0.50	0.66	0.44	0.77*
	(0.03)	(0.36)	(0.35)	(0.39)	(0.36)
Income	-0.11*	-0.32	0.20	-0.08	-0.44
	(0.04)	(0.51)	(0.51)	(0.57)	(0.53)
Female	0.04	-0.30	-0.04	-0.18	0.04
	(0.02)	(0.26)	(0.26)	(0.29)	(0.27)
Satisficing	0.15*	0.85*	0.83*	0.49	1.05*
	(0.04)	(0.39)	(0.39)	(0.42)	(0.40)
Sci. Know	-0.05	-0.17	-0.23	0.29	-0.02
	(0.04)	(0.51)	(0.51)	(0.59)	(0.53)
β_0	-0.04	-4.25*	-4.53*	-5.70*	-6.31*
	(0.09)	(1.18)	(1.18)	(1.34)	(1.30)
N	482.00	479.00	470.00	480.00	469.00

Note. Models used to produce Figure S1 swap the measure of MFW derived from the 2PL IRT method for the scale derived using GRM. Please refer to the caption for that Figure (and its accompanying Table) for additional information; as the models are otherwise analogous.

Table S18. Models Used to Produce Figure S2

	Anti-Exp.	Dr. (Immunity)	Dr. (Vax)	Sci (Immunity)	Sci (Vax.)	Policy Role
MFW	0.71*	4.75*	5.01*	4.96*	5.39*	0.12*
	(0.02)	(0.30)	(0.32)	(0.32)	(0.33)	(0.02)
Individualism	0.09*	1.10*	1.15*	1.42*	1.16*	-0.03*
	(0.02)	(0.19)	(0.20)	(0.21)	(0.21)	(0.02)
Democrat	-0.06*	0.26*	0.29*	0.39*	0.15	-0.05*
	(0.01)	(0.11)	(0.12)	(0.13)	(0.13)	(0.01)
Republican	0.02	0.34*	0.37*	0.42*	0.30*	-0.01
	(0.01)	(0.12)	(0.13)	(0.14)	(0.13)	(0.01)
Difficulty Seeing Dr.	0.06*	0.24*	0.43*	0.27*	0.49*	0.02*
	(0.01)	(0.09)	(0.09)	(0.10)	(0.09)	(0.01)
HS	-0.05*	-0.29	-0.36	-0.45	-0.19	0.03
	(0.02)	(0.26)	(0.26)	(0.27)	(0.29)	(0.02)
Some Coll.	-0.06*	-0.17	-0.28	-0.35	-0.01	0.01
	(0.02)	(0.26)	(0.27)	(0.28)	(0.29)	(0.02)
Coll.	-0.07*	-0.19	-0.30	-0.31	-0.02	-0.01
	(0.02)	(0.26)	(0.26)	(0.27)	(0.29)	(0.02)
Age	0.07*	-0.15	-0.36	-0.00	-0.72*	-0.10*
	(0.02)	(0.20)	(0.22)	(0.23)	(0.23)	(0.02)
Black	0.04*	0.28*	0.30*	0.20	0.10	0.02*
	(0.01)	(0.11)	(0.11)	(0.12)	(0.12)	(0.01)
Hisp.	0.02	0.38*	0.25*	0.31*	0.32*	-0.01
	(0.01)	(0.12)	(0.12)	(0.13)	(0.13)	(0.01)
Income	0.01	0.37*	0.37*	0.53*	0.45*	-0.04*
	(0.01)	(0.15)	(0.16)	(0.16)	(0.17)	(0.01)
Female	0.02*	-0.15	-0.18*	-0.42*	-0.36*	-0.01*
	(0.01)	(0.08)	(0.08)	(0.09)	(0.09)	(0.01)
Satisficing	0.15*	0.61*	0.40*	0.74*	0.48*	0.18*
	(0.02)	(0.18)	(0.19)	(0.19)	(0.19)	(0.02)
β_0	-0.01	-4.47*	-4.69*	-4.99*	-5.04*	0.20*
	(0.03)	(0.32)	(0.33)	(0.35)	(0.36)	(0.03)
N	4703.00	4701.00	4694.00	4700.00	4692.00	4703.00

Note. Models used to produce Figure S2 swap the measure of MFW derived from the 2PL IRT method for the scale derived using GRM. Please refer to the caption for that Figure (and its accompanying Table) for additional information; as the models are otherwise analogous.

Table S19. Models Used to Produce Figure S3

	Anti-Exp.	Dr. (Immunity)	Dr. (Vax)	Sci (Immunity)	Sci (Vax.)
MFW	0.30*	2.07*	1.85*	2.34*	2.28*
	(0.05)	(0.50)	(0.49)	(0.55)	(0.51)
Democrat	-0.05	0.98*	0.45	0.99*	0.77
	(0.03)	(0.40)	(0.38)	(0.45)	(0.40)
Republican	0.03	1.11*	0.52	0.95*	0.80*
	(0.03)	(0.42)	(0.38)	(0.47)	(0.41)
Individualism	0.07	-1.28	-0.83	-1.06	0.07
	(0.07)	(0.79)	(0.80)	(0.90)	(0.81)
Difficulty Seeing Dr.	0.05	0.75*	0.95*	1.07*	0.77*
	(0.03)	(0.28)	(0.27)	(0.30)	(0.28)
HS	0.06	-0.19	0.20	0.30	0.90
	(0.07)	(0.78)	(0.79)	(0.91)	(0.91)
Some Coll.	0.08	-0.34	-0.04	0.34	0.96
	(0.07)	(0.80)	(0.82)	(0.94)	(0.93)
Coll.	0.07	0.20	0.58	0.90	1.52
	(0.07)	(0.79)	(0.80)	(0.92)	(0.92)
Age	0.04	0.61	0.52	-0.23	-0.09
	(0.05)	(0.58)	(0.59)	(0.67)	(0.61)
Black	0.04	0.89*	0.56	0.56	0.49
	(0.04)	(0.36)	(0.36)	(0.39)	(0.37)
Hisp.	0.04	0.63	0.78*	0.63	0.87*
	(0.03)	(0.35)	(0.34)	(0.36)	(0.34)
Income	-0.13*	-0.50	0.05	-0.28	-0.56
	(0.04)	(0.50)	(0.49)	(0.55)	(0.51)
Female	0.02	-0.36	-0.12	-0.25	-0.07
	(0.02)	(0.25)	(0.25)	(0.28)	(0.26)
Satisficing	0.17*	0.82*	0.83*	0.49	1.01*
	(0.04)	(0.39)	(0.38)	(0.40)	(0.39)
Sci. Know	-0.11*	-0.57	-0.63	-0.22	-0.46
	(0.04)	(0.49)	(0.49)	(0.55)	(0.50)
β_0	0.23*	-2.53*	-2.80*	-3.77*	-4.35*
	(0.09)	(1.09)	(1.08)	(1.26)	(1.20)
N	482.00	479.00	470.00	480.00	469.00

Note. Models used to produce Figure S3 swap the measure of MFW derived from the 2PL IRT method for the short form scale described in the text. Please refer to the caption for that Figure (and its accompanying Table) for additional information; as the models are otherwise analogous.

Table S20. Models Used to Produce Figure S4

	Anti-Exp.	Dr. (Immunity)	Dr. (Vax)	Sci (Immunity)	Sci (Vax.)	Policy Role
MFW	0.36*	2.08*	2.27*	2.39*	2.62*	0.10*
	(0.01)	(0.15)	(0.16)	(0.17)	(0.17)	(0.01)
Individualism	0.13*	1.38*	1.44*	1.70*	1.46*	-0.04*
	(0.02)	(0.19)	(0.20)	(0.21)	(0.21)	(0.02)
Democrat	-0.06*	0.26*	0.29*	0.39*	0.16	-0.05*
	(0.01)	(0.11)	(0.12)	(0.13)	(0.13)	(0.01)
Republican	0.02	0.37*	0.40*	0.45*	0.32*	-0.01
	(0.01)	(0.12)	(0.13)	(0.14)	(0.13)	(0.01)
Difficulty Seeing Dr.	0.06*	0.28*	0.46*	0.29*	0.50*	0.02*
	(0.01)	(0.09)	(0.09)	(0.10)	(0.09)	(0.01)
HS	-0.06*	-0.33	-0.41	-0.49	-0.24	0.02
	(0.02)	(0.25)	(0.26)	(0.27)	(0.28)	(0.02)
Some Coll.	-0.07*	-0.28	-0.39	-0.45	-0.12	0.01
	(0.02)	(0.26)	(0.26)	(0.28)	(0.29)	(0.02)
Coll.	-0.08*	-0.28	-0.39	-0.39	-0.11	-0.01
	(0.02)	(0.25)	(0.26)	(0.27)	(0.28)	(0.02)
Age	0.05*	-0.33	-0.54*	-0.15	-0.86*	-0.10*
	(0.02)	(0.20)	(0.21)	(0.22)	(0.23)	(0.02)
Black	0.05*	0.39*	0.40*	0.30*	0.20	0.02*
	(0.01)	(0.11)	(0.11)	(0.12)	(0.12)	(0.01)
Hisp.	0.02*	0.40*	0.27*	0.33*	0.34*	-0.01
	(0.01)	(0.11)	(0.12)	(0.12)	(0.12)	(0.01)
Income	-0.00	0.31*	0.31*	0.47*	0.38*	-0.04*
	(0.01)	(0.15)	(0.15)	(0.16)	(0.16)	(0.01)
Female	0.02*	-0.17*	-0.20*	-0.43*	-0.37*	-0.01*
	(0.01)	(0.08)	(0.08)	(0.09)	(0.09)	(0.01)
Satisficing	0.14*	0.53*	0.31	0.62*	0.37	0.17*
	(0.02)	(0.18)	(0.19)	(0.19)	(0.19)	(0.02)
β_0	0.19*	-3.00*	-3.17*	-3.58*	-3.51*	0.22*
	(0.03)	(0.29)	(0.30)	(0.31)	(0.32)	(0.02)
N	4703.00	4701.00	4694.00	4700.00	4692.00	4703.00

Note. Models used to produce Figure S1 swap the measure of MFW derived from the 2PL IRT method for the scale derived using GRM. Please refer to the caption for that Figure (and its accompanying Table) for additional information; as the models are otherwise analogous.

Summary Statistics

Table S21. Study 2 Summary Statistics (for Variables NOT Presented in Table M1)

Variable	Mean	SD	Min	Max	N
Medical Folk Wisdom Scale	0.49	0.23	0	1	509
Know. Assessment (Med. Dr., Disease)	0.23	0.42	0	1	494
Know. Assessment (Scientists, Disease)	0.17	0.38	0	1	495
Know. Assessment (Med. Dr., Vaccines)	0.23	0.42	0	1	484
Know. Assessment (Scientists, Vaccines)	0.22	0.42	0	1	483
Anti-Expert Attitudes	0.43	0.27	0	1	497
Avoid Public Places When Sick	1.88	0.97	0	3	495
Wash Hands	2.54	0.76	0	3	497
Wear Seatbelt	2.66	0.72	0	3	496
Visit Dr. When Sick	1.58	0.96	0	3	497
Democrat Self-ID	0.42	0.49	0	1	509
Republican Self-ID	0.39	0.49	0	1	509
Individualism (Cult. Cog.)	0.57	0.17	0	1	497
Trouble Accessing Dr.	0.22	0.42	0	1	497
Good Health Indicator	0.78	0.41	0	1	496
Satisficing Indicator	0.12	0.32	0	1	509

Note. Sample summary statistics for all independent and dependent variables *not* summarized in the Online Methods. Information about how all of these items were measured can be found in the Online Methods section, and full question wording can be found in the Question Wording section of the Supplementary Materials.

Table S22. Study 2 Summary Statistics (for Variables NOT Presented in Table M1)

Variable	Mean	SD	Min	Max	N
Medical Folk Wisdom Scale	0.47	0.23	0	1	4998
Know. Assessment (Med. Dr., Disease)	0.22	0.41	0	1	4887
Know. Assessment (Scientists, Disease)	0.18	0.38	0	1	4886
Know. Assessment (Med. Dr., Vaccines)	0.2	0.4	0	1	4880
Know. Assessment (Scientists, Vaccines)	0.18	0.38	0	1	4878
Anti-Expert Attitudes	0.37	0.27	0	1	4889
Oppose Role Exp. Play in HP	0.18	0.23	0	1	4889
Avoid Public Places When Sick	1.87	0.93	0	3	4889
Wash Hands	2.58	0.69	0	3	4888
Wear Seatbelt	2.69	0.68	0	3	4889
Visit Dr. When Sick	1.51	0.94	0	3	4889
Democrat Self-ID	0.46	0.5	0	1	4998
Republican Self-ID	0.36	0.48	0	1	4998
Self Enhancement (Individualism)	0.46	0.23	0	1	4887
Trouble Accessing Dr.	0.24	0.43	0	1	4825
Good Health Indicator	0.79	0.4	0	1	4998
Satisficing Indicator	0.05	0.23	0	1	4998

Note. Sample summary statistics for all independent and dependent variables *not* summarized in the Online Methods. Information about how all of these items were measured can be found in the Online Methods section, and full question wording can be found in the Question Wording section of the Supplementary Materials.
