# Supplementary material for: The pervasiveness and policy consequences of medical folk wisdom in the U.S
Source: Sci Rep. 2020 Jul 1;10:10722. doi: 10.1038/s41598-020-67744-6 (PMC7329847; doi:10.1038/s41598-020-67744-6)
Supplement: Supplementary file 2 — Supplementary file2 (DOCX 24 kb) [file 41598_2020_67744_MOESM2_ESM.docx]

The Pervasiveness and Policy Consequences of Medical Folk Wisdom in the U.S.

Matthew Motta, PhD

Assistant Professor

Department of Political Science

Oklahoma State University

matthew.motta@okstate.edu

Timothy Callaghan, PhD

Assistant Professor

Department of Health Policy & Management

Texas A&M University

[callaghan@tamu.edu](mailto:callaghan@tamu.edu)

**Table A1. Fully Referenced Supplementary Version of Table 1 (in-text)**

| **Folk Theory** | **Evidence** | **% Endorsed** |
| --- | --- | --- |
| “Exposure to cold weather can cause you to catch a cold.” | Exposure to rhinoviruses, *irrespective of weather conditions*, causes people to catch colds. [1] People are to catch colds in winter [2], but that’s because we spend more time indoors [3].  Cold weather is associated with decreased immune responsiveness in mice [4], and more favorable survival conditions for viruses [5], but there is little evidence that exposure to cold weather | 49% (S1), 46% (S2) |
| “Consuming more than the daily recommended amount of vitamin C can prevent illnesses like influenza and the common cold.” | A recent meta-analysis found that vitamin C, and excess consumption of it (e.g., via supplements), does not reduce incidence of the common cold. [6] | 55% (S1), 49% (S2) |
| “Eating chicken soup can help people recover from illnesses more quickly.” | Eating chicken soup may reduce respiratory inflammation, but it has no known medicinal benefits regarding its ability to fight infections [7]. | 66% (S1), 63% (S2) |
| “Not washing one’s hands can help increase immunity to disease” | Hand washing is an effective way [8] to prevent the transfer of germs that cause disease. Poor hygiene and not washing one’s hands does not [9-10] act as a natural form of inoculation. | 38% (S1), 37% (S2) |
| “Taking multivitamins daily can help prevent catching illnesses like the common cold.” | Neither vitamin C [11], nor multivitamins [12] have been shown to reduce cold incidence. Some studies [13] find beneficial effects of Zinc when taken immediately at the *outset* of a cold, but with the important caveat [14] that (1) these claims are disputed, and (2) Zinc does not *prevent* colds. | 62% (S1), 72% (S2) |
| “Carbonated drinks, like ginger ale, can cure stomach aches” | Ginger in its natural form can have health benefits [15]. However, there is not evidence [16] that sugar sweetened beverages like ginger ale offer much relief. | 63% (S1), 62% (S2) |
| “Women cannot become pregnant by having sex during menstruation (or “on their period”).” | While women may be less likely [17] to become pregnant while having sex during menstruation, sex during menstruation can result in pregnancy [18]. | 30% (S1), 28% (S2) |
| “White spots on one’s fingernails are indicative of not consuming enough Vitamin C.” | Although white spots (known as leukonychia) can occur for a number of different reasons, the most common is minor injury [19] to the fingernail area. Vitamin deficiencies are not thought [20] to be responsible for leukonychia. | 58% (S1), 54% (S2) |
| “Showering after sex is an effective way to prevent pregnancy.” | Showering after sex will not [21] help prevent pregnancy. In fact, research suggests that the act of rinsing the vagina after sex may actually push sperm further up through the cervix [22]. That said, cleaning yourself after sex can protect from some infections like UTIs [23]. | 15% (S1), 13% (S2) |
| “Cracking one’s knuckles can cause arthritis.” | Consistent medical research [24-25] suggests that cracking knuckles is not correlated with degenerative changes associated with arthritis. | 49% (S1), 47% (S2) |
| “Not eating when one has a fever (sometimes called "starving a fever") can reduce the amount of time it takes to recover” | While the idea of fasting to stop a fever dates back to the 1500s, it is not based in scientific consensus [26-27]. Fevers increase body temperature and metabolism and thus burn more calories. Eating can actually help to replace the calories that are lost due to the fever.  That said, research in mice [28] suggests the importance of eating may depend on the root cause of the fever, with it proving beneficial for influenza but detrimental for bacterial infections. | 37% (S1), 33% (S2) |

1. Klein, S. (2015). Can You Really Get a Cold from Going Outside with Wet Hair? (Interview with Dr. Pritish Tosh, Mayo Clinic) *The Huffington Post*. <https://www.huffpost.com/entry/cold-wet-hair-germs_n_6739144>
2. Ikäheimo, T., Jaakkola, K., Jokelainen, J., Saukkoriipi, A., Roivainen, M., Juvonen, R., ... & Jaakkola, J. (2016). A decrease in temperature and humidity precedes human rhinovirus infections in a cold climate. *Viruses*, *8*(9), 244.
3. Heaney, K. (2017). It’s Suddenly Cold Out. Am I Going to Get Sick? *The Atlantic.* <https://www.theatlantic.com/health/archive/2017/12/can-temperature-changes-make-you-sick/547760/>
4. Foxman, E. F., Storer, J. A., Fitzgerald, M. E., Wasik, B. R., Hou, L., Zhao, H., ... & Iwasaki, A. (2015). Temperature-dependent innate defense against the common cold virus limits viral replication at warm temperature in mouse airway cells. *Proceedings of the National Academy of Sciences*, *112*(3), 827-832.
5. Jacobs, S. E., Lamson, D. M., George, K. S., & Walsh, T. J. (2013). Human rhinoviruses. *Clinical microbiology reviews*, *26*(1), 135-162.
6. [h](https://journals.plos.org/plosmedicine/article/file?id=10.1371/journal.pmed.0020168&type=printable)Hemilä, H., & Chalker, E. (2013). Vitamin C for preventing and treating the common cold. *Cochrane database of systematic reviews*, (1).
7. Rennard, B. O., Ertl, R. F., Gossman, G. L., Robbins, R. A., & Rennard, S. I. (2000). Chicken soup inhibits neutrophil chemotaxis in vitro. *Chest*, *118*(4), 1150-1157.
8. Center for Disease Control (2018). *Why Wash Your Hands?* <https://www.cdc.gov/handwashing/why-handwashing.html>
9. Baird, C. (2013) How Does Not Washing Your Hands Make you a Healthier Person? <http://wtamu.edu/~cbaird/sq/2013/05/03/how-does-not-washing-your-hands-make-you-a-healthier-person/>
10. Bowerman, M. (2017). What Would Happen if you Stopped Washing your Hands? *USA Today.* <https://www.usatoday.com/story/news/nation-now/2017/05/01/what-would-happen-if-you-stopped-washing-your-hands/99585016/>
11. Harvard Health Letter Editorial Team. (2017). Can Vitamin C Prevent a Cold? *Harvard Health Letters.* <https://www.health.harvard.edu/cold-and-flu/can-vitamin-c-prevent-a-cold>
12. Graat, J. M., Schouten, E. G., & Kok, F. J. (2002). Effect of daily vitamin E and multivitamin-mineral supplementation on acute respiratory tract infections in elderly persons: a randomized controlled trial. *Jama*, *288*(6), 715-721.
13. Hemilä, H. (2017). Zinc lozenges and the common cold: a meta-analysis comparing zinc acetate and zinc gluconate, and the role of zinc dosage. *JRSM open*, *8*(5), 2054270417694291.
14. Bauer, B.A. (2017). Will Taking Zinc for Colds Make my Colds Go Away Faster? <https://www.mayoclinic.org/diseases-conditions/common-cold/expert-answers/zinc-for-colds/faq-20057769>
15. Singletary, K. (2010). Ginger: an overview of health benefits. *Nutrition Today*, *45*(4), 171-183.
16. Pathak, N. (2018) Best and Worst Drinks When You Have a Cold. <https://symptoms.webmd.com/cold-flu-map/how-to-stay-hydrated-when-youre-sick>
17. American Pregnancy Organization. (2019). Can You Get Pregnant On Your Period? <https://americanpregnancy.org/getting-pregnant/can-get-pregnant-period/>
18. Anzilotti, A.W. (2018) Can a Girl Get Pregnant if She Has Sex on Her Period? <https://kidshealth.org/en/teens/sex-during-period.html>
19. Cecil, R. L. F., Goldman, L., & Schafer, A. I. (2012). *Goldman's Cecil Medicine, Expert Consult Premium Edition--Enhanced Online Features and Print, Single Volume, 24: Goldman's Cecil Medicine* (Vol. 1). Elsevier Health Sciences.
20. Morgan, Z., & Wickett, H. (2011). Leukonychia on finger nails as a marker of calcium and/or zinc deficiency. *Journal of Human Nutrition and Dietetics*, *24*(3), 294-295.
21. Ask Alice Editors at Columbia University (2014). Does Washing Immediately After Intercourse Prevent Pregnancy? )<https://goaskalice.columbia.edu/answered-questions/does-washing-immediately-after-intercourse-prevent-pregnancy>
22. Planned Parenthood (2010). Can a Girl Get Pregnant if She Washes Out Her Vagina After Sex? <https://www.plannedparenthood.org/learn/teens/ask-experts/after-sex-without-a-condom-if-the-female-washes-out-her-vagina-with-hot-water-and-soap-can-she-still-get-pregnant>
23. DerSarkissian, C. (2019). Things You Should and Shouldn’t Do After Sex. <https://www.webmd.com/sex-relationships/ss/slideshow-sexual-hygiene>
24. Swezey, R. L., & Swezey, S. E. (1975). The consequences of habitual knuckle cracking. *Western Journal of Medicine*, *122*(5), 377.
25. DeWeber, K., Olszewski, M., & Ortolano, R. (2011). Knuckle cracking and hand osteoarthritis. *J Am Board Fam Med*, *24*(2), 169-174.
26. Fishetti, M. (2014) Fact or Fiction? Feed a Cold, Starve a Sever. *Scientific American.* <https://www.scientificamerican.com/article/fact-or-fiction-feed-a-cold/>
27. O’Conner, A. (2007). The Claim: Starve a Cold, Feed a Fever. *The New York Times.* <https://www.nytimes.com/2007/02/13/health/13real.html>
28. Wang, A., Huen, S. C., Luan, H. H., Yu, S., Zhang, C., Gallezot, J. D., ... & Medzhitov, R. (2016). Opposing effects of fasting metabolism on tissue tolerance in bacterial and viral inflammation. *Cell*, *166*(6), 1512-1525.
